# Success with incrementally faster times to endovascular therapy (SWIFT-EVT): A systematic review and meta-analysis

**DOI:** 10.1016/j.jstrokecerebrovasdis.2024.107964

**Published:** 2024-08-23

**Authors:** Brittney Legere, Ahmed Mohamed, Salah Elsherif, Razan Saqqur, David Schoenfeld, Anna M. Slebonick, Michael McCartin, James Price, Kori S. Zachrison, Jonathan A. Edlow, Maher Saqqur, Ashfaq Shuaib, Stephen H. Thomas

**Affiliations:** a Department of Applied Human Sciences, University of Guelph, Guelph, Ontario, Canada; b Department of Physiology, Temerty Faculty of Medicine, University of Toronto, Ontario, Canada; c Department of Health Sciences, Queens University, Kingston, Ontario, Canada; d Department of Health, University of Waterloo, Waterloo, Ontario, Canada; e Department of Emergency Medicine, Beth Israel Deaconess Medical Center & Harvard Medical School, Boston, MA, USA; f Drexel University School of Medicine, Philadelphia, PA, USA; g Section of Emergency Medicine, University of Chicago, Chicago, IL, USA; h Department of Emergency Medicine, Cambridge University NHS Trust, Cambridge, UK; i Department of Emergency Medicine, Massachusetts General Hospital & Harvard Medical School, Boston, MA, USA; j Department of Neurology, University of Toronto, Mississauga, ON, Canada; k Department of Neurology, University of Alberta, Edmonton, AB, Canada; l Blizard Institute for Neuroscience, Surgery, & Trauma, Barts & The London School of Medicine, London, UK

**Keywords:** Stroke, Endovascular therapy, Treatment time, Emergency medical services, Helicopter transport

## Abstract

**Background:**

A major systematic review and meta-analysis assessing trial data through 2014 (the Highly Effective Reperfusion Evaluated in Multiple Endovascular Stroke Trials, HERMES) demonstrated that particularly over the initial six hours of acute ischemic stroke (AIS), rapid performance of endovascular therapy (EVT) markedly improves outcomes. The current analysis, Success with Incrementally Faster Times to EVT (SWIFT-EVT), aimed to provide an updated metric summarizing latest estimates for modified Rankin Scale (mRS) improvements accrued by streamlining time to EVT.

**Methods:**

A systematic review and meta-analysis was conducted using electronic databases. Eligible studies reported a time-benefit slope with times from AIS onset (or time last known normal) to EVT commencement; the predictor was onset-to-groin (OTG) time. Primary and secondary outcomes were 90-day functional independence (mRS 0-2) and 90-day excellent function (mRS 0-1), respectively.

**Results:**

Five studies were included. Results showed increased change of good outcome with each hour of pre-EVT time savings for mRS 0-2 for 0-270’ (OR 1.25, 95 % CI 1.16-1.35, *I*^2^ 40 %) and 271-360’ time frame (1.22, 95 % CI 1.12-1.33, *I*^2^ 58 %). For the studies assessing mRS 0-1, estimates were found appropriate for both the 0-270’ time frame (OR 1.34, 95 % CI 1.19-1.51, *I*^2^ 27 %) and the 271-360’ time frame (OR 1.20, 95 % CI 1.03-1.38, *I*^2^ 60 %).

**Conclusions:**

Each hour saved from AIS onset to EVT start is associated with a 22-25 % increased odds of achieving functional independence, a useful metric to inform patient-specific and systems planning decisions.

## Background

Acute management decisions in patients with acute ischemic stroke (AIS) are highly time-critical. This urgency is in large part due to clear benefits of faster initiation of reperfusion therapy, such as significantly lower rates of disability and improved functional independence at 90 days.^1^ Speed to endovascular therapy (EVT) is so crucial that a citizens proximity to comprehensive stroke centers (CSCs) equipped for EVT is used as an indicator of access to high-quality medical care.^2^

A 2016 systematic review and meta-analysis from the HERMES^3^ group assessed five 2015 studies^4–8^ reporting 89 international sites’ experience with EVT. The HERMES results showed increasingly improved patient outcomes based on streamlining intervals from symptom onset to EVT commencement ("symptom to groin time"). HERMES found significant time-savings benefits throughout a symptom-to-groin time frame extending over seven hours from stroke onset.^3^ A follow-up analysis from HERMES extended the results to link time savings to successful reperfusion as measured by the modified thrombolysis in cerebral infarction (mTICI) scale.^9^

Since 2014, the last year included in the 2016 HERMES report, AIS management has evolved. One such evolution relates to patient transport. The critical role of emergency medical services (EMS) care and transport decisions in EVT access is important when considering timeliness of thrombolysis through improved organization of stroke systems. EMS triage and transport decisions are increasingly critical to streamlining movement of AIS patients with large-vessel occlusion (LVO) to EVT-capable CSCs.^1,10,11^ Reducing these transport times is important: each minute saved in reperfusion yields an additional 4-7 days’ of healthy life and saves millions of neurons.^12,13^

A 2016 meta-analysis^3^ demonstrated EVT effectiveness and time-savings importance through seven hours after symptom onset. Meta-analysis has also suggested that for patients with anterior LVO ischemic stroke, the number needed to treat (NNT) is only 2.6, for a one-point improvement in modified Rankin Scale (mRS).^14^ Additional studies have also provided evidence for the effects of time to EVT on outcome in the last seven years.^15–19^ However, no recent systematic reviews have quantified patient outcomes based on time intervals and the time saving benefit of EVT. The current study, Success With Incrementally Faster Times to EVT (SWIFT EVT), aimed to calculate precise patient-centered outcome benefits for incremental pre-EVT time savings within six hours of stroke onset. The six-hour window was selected as it is a broadly accepted (and guideline-based^20^) window for prioritizing time to EVT for LVO AIS.^21^

## Methods

### Design

This systematic review and meta-analysis was conducted in accordance with the PRISMA guidelines.^22^ All data arose from published sources in the medical literature and there was no patient or public involvement, thus ethics board approval was not required.

The review was registered with the UK’s National Institute for Health and Care Research International Prospective Register of Systematic Reviews (PROSPERO, registration number 483826).

## Search strategy

*A priori* eligibility criteria for screening studies for potential inclusion were: diagnosis (ischemic stroke), intervention (EVT), and endpoint of mRS in the “functional independence” range of 0-2. In order to avoid overlap with data analyzed for the 2016 meta-analysis,^3^ only studies including cases focusing on the year 2014 onward were eligible for inclusion. HERMES assessed studies from 5 different trials. In each trial they accrued patients through some part of 2014. Studies that included patients from the same registry, but who were not included in the time frame covered by HERMES, were eligible for inclusion in this analysis

In addition to excluding studies with overlapping enrollments,^3^ we also excluded studies that examined only a particular severity subset of EVT cases.^23,24^ This decision was made *a priori* based on two factors. First, while ongoing work suggests EVT utility even in severe strokes,^21,25^ we believed it highly likely that effect estimates for EVT time savings would be different at the extremes of severity defined by scores such as Alberta Stroke Program Early CT Score (ASPECTS). Second, our aim was to provide broad-based, patient-relevant information. Comprehensive imaging results may not be known at the time of transport decisions. Since advanced imaging is neither required nor typically available at the time of transport decision-making,^26^ we wished to generate a pooled time-savings estimate that was not restricted to cases with advanced imaging results.

Our literature search strategy employed variations of three medical subject terms: EVT, time, and outcome. The search used the National Library of Medicine (PubMed.gov) Medline site, EMBASE, and Google-Scholar (for gray literature). In addition, the reference lists of selected trials were assessed to identify further eligible studies. Detailed search information is reported in the Supplement.

At least two authors reviewed title and abstract for all records identified in the initial search. Any disagreements in the initial eligibility recommendation were adjudicated by consensus and discussion with a third author. Studies thought likely eligible after title/abstract review, as well as studies in which there was disagreement as to possible eligibility, were reviewed in full-text version. Review of studies’ full-text included any supplemental article information (e.g. on-line results).

Eligible studies needed to describe an association (including a null association) between time-savings and patient outcomes. This review set as the time window (from symptom onset or last-known-normal) of six hours to EVT start time, to define the time window of focus for calculating our effect estimates. The six-hour time was defined *a priori* to meet widely accepted windows for EVT benefit.^27,21^ For studies that evaluated EVT time-savings benefits for longer time frames, this analysis focused on the study results reported up to six hours.

### Endpoints

Studies meeting initial eligibility criteria were reviewed against additional pre-defined eligibility criteria related to endpoints. We included only studies that reported on our primary endpoint of functional independence (mRS of 0-2). Optional secondary endpoints included excellent outcome (mRS 0-1), mortality, and level of occlusion.

### Evaluation of study quality

Study quality was assessed using standardized tools. With regard to our endpoints of interest, we applied the Risk of Bias in Non-randomized Studies of Interventions (ROBINS-I)^28^ to each study, incorporating principles of evidentiary grading as outlined in Grading of Recommendations, Assessment, Development, and Evaluations (GRADE).^29^ The ROBINS-I approach we used was modified to focus on the endpoints applicable to SWIFT-EVT.

### Statistical analysis and reporting

To derive time-related EVT benefits, the first step was collection of each study’s reported incremental benefits in the metric of adjusted odds ratio (OR) and its 95 % confidence interval (CI). Study-reported absolute risk difference (ARD) was also recorded, if the reported ARD was an adjusted metric.

Translation of the OR to the absolute effect measure (ARD) and NNT was executed using methods and formulae outlined by the Cochrane Group.^29,30^ Calculation of NNT depends on both the estimated baseline “risk” of the endpoint of interest (either functional independence or mRS 0-1) and the time frame (0-270’ or 271-360’). Study planning called for plotting of sample NNT based on baseline 90-day functional independence of 50 %.

All other analysis and plotting were executed using Stata (Version 18MP, Stata Corp, College Station TX). Significance was set at the *p* <.05 level.

To assess the primary and secondary endpoints, we executed random effects modeling to generate a pooled effect estimate for benefits accrued by each 60 min incremental pre-EVT time savings over our study window of 0-360 min. After the number of studies (N) was determined to be small (five studies for primary endpoint, three studies for secondary endpoint), we employed a Hartung-Knapp-Sidik-Jonkman (HKSJ) approach recommended for small-*N* meta-analysis.^31^ Sensitivity to model selection was assessed by re-executing meta-analysis using a second random effects approach, the DerSimonian-Laird (DL) model. A third modeling approach, fixed-effect modeling, was executed solely as an informal indicator of study heterogeneity (*i.e.* to determine whether results were markedly different from random effects results).

Formal heterogeneity and small-study bias (including publication bias) were evaluated using *I*^2^ (with 30-60 % representing “potentially moderate”^32^ heterogeneity), Galbraith plotting, and funnel plots with trim-and-fill (imputed study) analysis. Sensitivity analyses included omitted-study plotting and cumulative meta-analysis. Meta-regression and prediction intervals were planned in the event the analysis accrued the minimum requirement of ten studies.^32^

## Results

### Identified records and characteristics of individual studies

The search strategy identified 14,092 records. After screening by title and/or abstract, 120 records were reviewed in full text, before identifying a total of *N* = 5 studies meeting eligibility criteria. Details on search results are provided in the Supplement.

Characteristics of the assessed studies are outlined in detail in Table 1. Further information regarding the assessed studies is found in the Supplement.

Four of the studies^16–19^ came from large national or international registries, whereas one^15^ came from a single (Italian) region. The large-registry studies all had approximately a thousand cases or more, thus yielding reasonable precision. The largest study’s authors^17^ pointed out that their large n (6,756) increased sensitivity to non-linearity in the onset to groin (OTG) outcome curve.

The Jahan study^17^ did in fact identify a “knot” in spline regression analysis, with the change in slope identified at approximately the 270 min (4.5 h) mark. As such, Jahan reported two different coefficients for the association between OTG and each of the three endpoints we assessed: mRS 0-2, mRS 0-1, and mortality.

The finding of non-linearity in the largest study influenced our own results presentation. It was judged inappropriate to simply apply a single effect estimate from Jahan^17^ when those authors reported two different slopes to the curve relating time-savings and outcome. While we judged the difference in slopes to be of marginal importance to either individual patient decision-making or systems planning, we believe this judgment should be left to readers. Therefore, our results presentation includes estimates for an “early” time frame of 0-270 min and a “later” time frame of 271-360 min. Results for the studies other than Jahan^17^ are replicated in both time frames, but the Jahan results contributing to the overall effect estimates were split into the initial 270 min (4.5 h) and the 271-360’ time frame (4.5 to 6 h).

### Primary endpoint: functional independence (mRS of 0-2) as a function of OTG

**Fig. 1.**
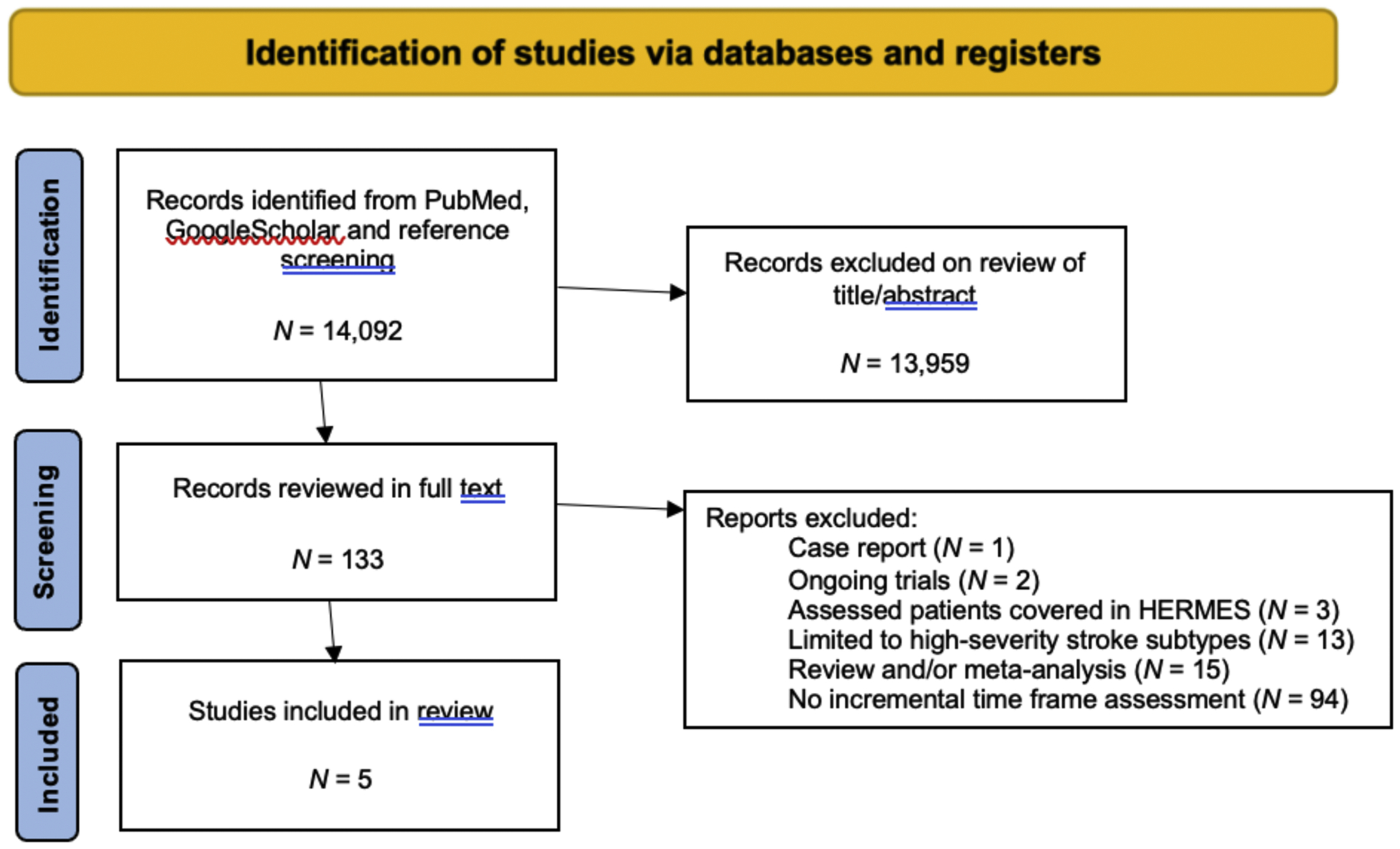
PRISMA flow diagram demonstrating study selection for data included in the meta-analysis.

**Fig. 2.**
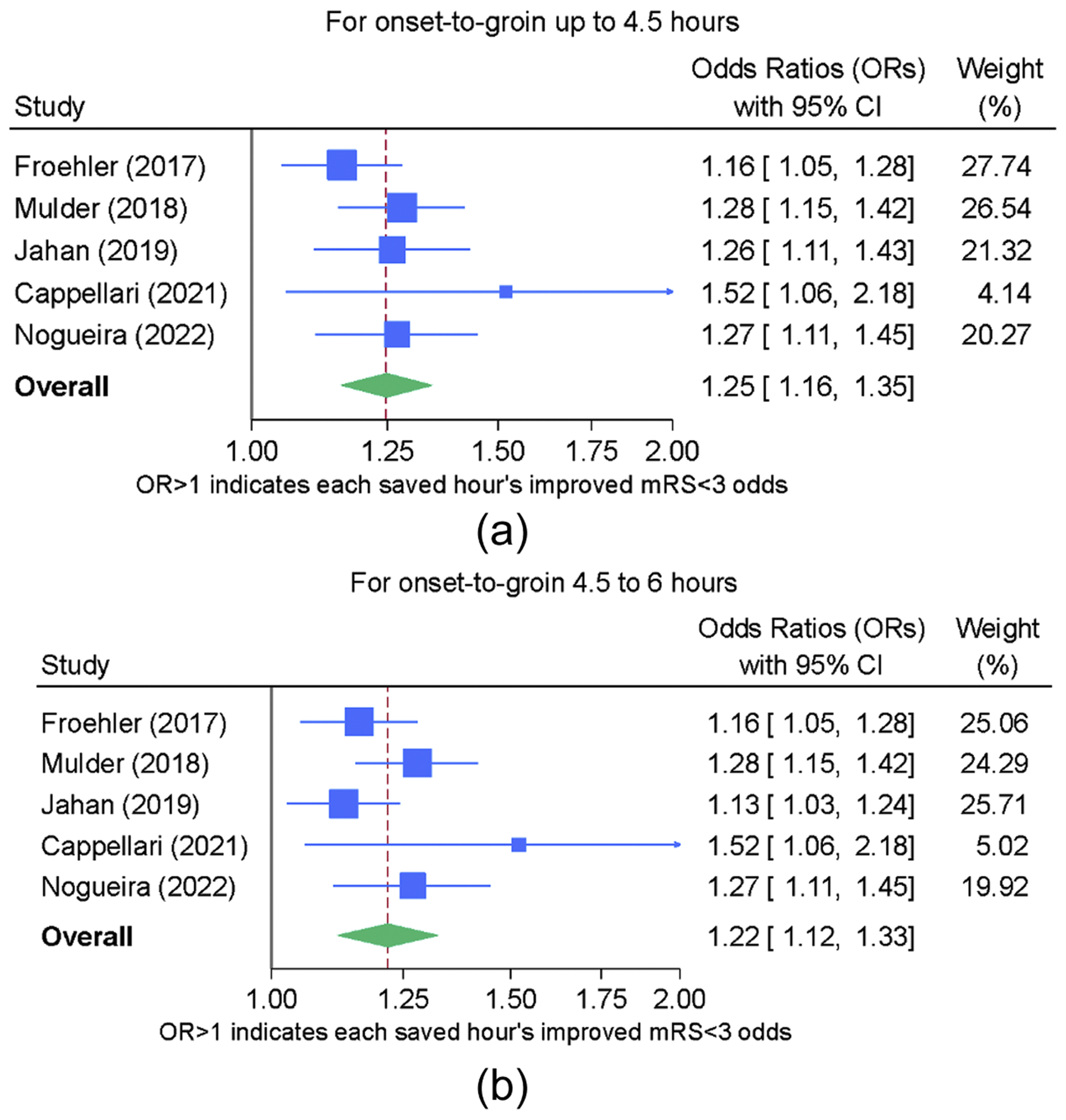
a. Forest plot for primary endpoint of each hour’s time savings and incremental gain in functional independence for 0-270’ time frame b. Forest plot for primary endpoint of each hour’s time savings and incremental gain in functional independence for 271-360’ time frame.

The meta-analysis results for functional independence are depicted in Figs. 2a and 1b. The *I*^2^ values for both the initial time frame of 0-270’ (*I*^2^ 40 %) and the later time frame of 271-360’ (*I*^2^ 58 %) indicated potential for moderate heterogeneity. For both time frames, concerning heterogeneity was judged unlikely based on non-significant Cochrane’s Q, a reassuring Galbraith graph, and on the fact that fixed-effect modeling generated similar effect estimates to those from HKSJ random effects modeling. Detailed results on heterogeneity analysis (for all endpoints) are provided in the Supplement.

Given the conclusion that marked heterogeneity did not preclude generation of a pooled effect estimate for time-savings influence on the functional independence outcome, the forest plot shown in Fig. 2 provides the overall OR estimate with its 95 % CI. For each hour saved within the time frame of 0-270’, the odds of achieving functional independence improve by 25 % and within the later time frame of 271-360’ the time-savings benefit is 22 %.

Adjusted ARR for functional independence for each hour of time saved was reported by three studies. Froehler^16^ and Mulder^18^ reported similar (5.3-5.5 %) and linear ARR over the entire six-hour window from AIS onset to EVT start. Jahan^17^ reported only an ARR (4.1 %) for the 0-270’ time period.

### Secondary endpoint: excellent outcome (mRS of 0-1) as a function of OTG

**Fig. 3.**
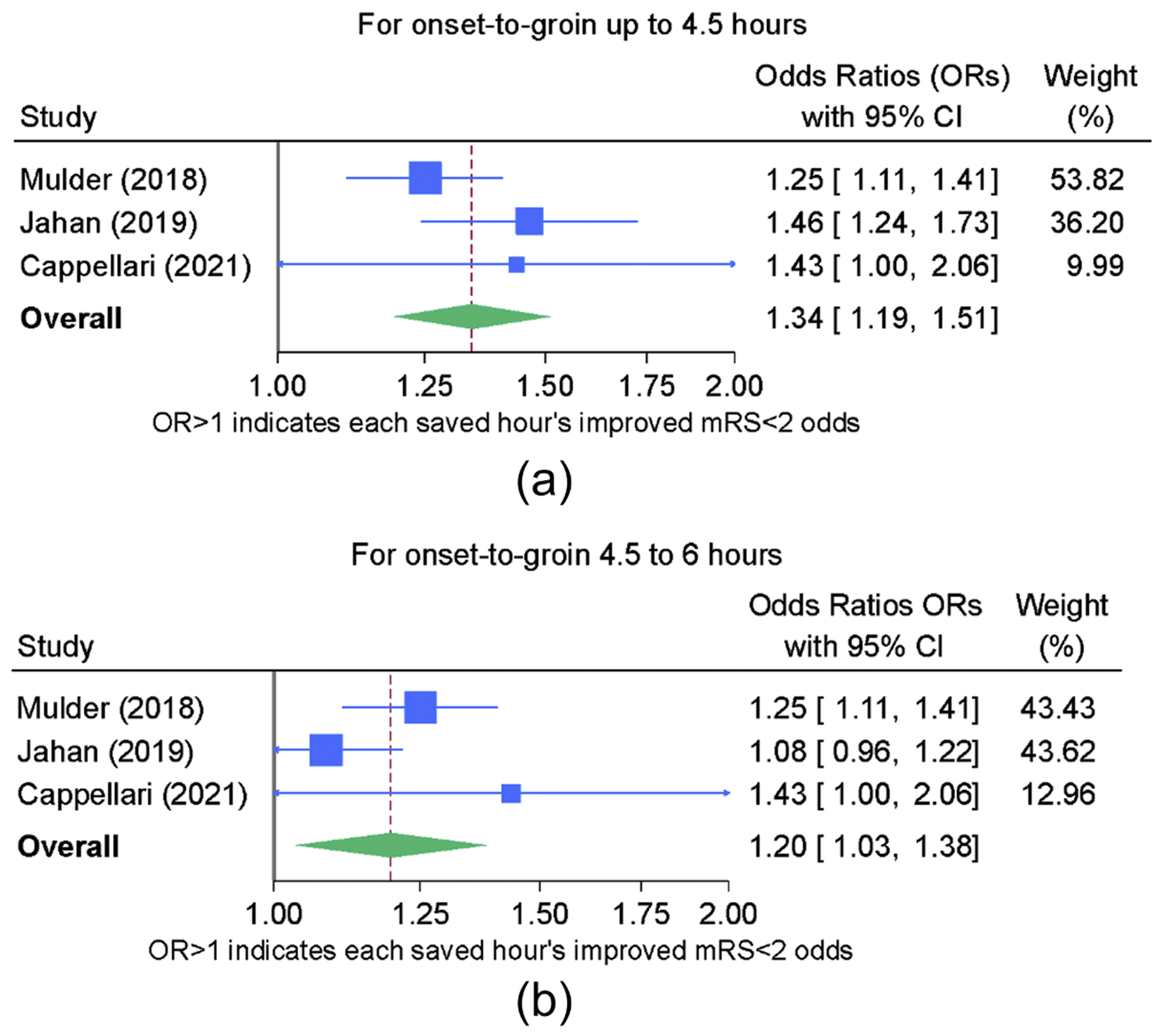
a. Forest plot for secondary endpoint of each hour’s time savings and incremental gain in mRS 0-1 for 0-270’ time frame b. Forest plot for secondary endpoint of each hour’s time savings and incremental gain in mRS 0-1 for 271-360’ time frame.

The results for mRS 0-1 are depicted in Fig. 3a and 3b. The *I*^2^ values for both the initial time frame of 0-270’ (*I*^2^ 27 %) and the later time frame of 271-360’ (*I*^2^ 60 %) indicated potential for moderate heterogeneity. As was the case with the primary endpoint, Cochrane’s Q, a reassuring Galbraith graph, and fixed-effects modeling provided support for a finding that heterogeneity was not present to a degree precluding generation of a pooled effect estimate.

Given the judgment that marked heterogeneity did not preclude generation of a pooled effect estimate for time-savings influence on the mRS 0-1 outcome, the forest plot shown in Fig. 3a and 3b provides the overall OR estimate. For each hour saved within the time frame of 0-270’, the odds of achieving mRS 0-1 improve by 34 % and within the later time frame of 271-360’ the time-savings benefit drops to 20 %.

### Additional meta-analysis results

Detailed assessment of the meta-analysis findings for the primary and secondary endpoints is found in the Supplement. The findings for the primary and secondary endpoints were not sensitive to model selection. Neither Galbraith graphing nor funnel plotting identified concerns for small-study (including publication) bias or outlier study influence. Trim-fill analysis yielded zero imputed studies for either primary or secondary endpoints, for either the early or the later time frame. Cumulative meta-analysis identified stabilization in effect estimates with addition of studies with increasing precision. There were insufficient studies to execute advanced techniques such as meta-regression or calculation of prediction intervals.

Mortality was not a primary endpoint for SWIFT-EVT, but three studies^15,17,18^ did report on a time-savings and mortality relationship. Analysis of these results was limited by sufficiently high heterogeneity (*I*^2^ 94 % for 0-270’ time frame, *I*^2^ 88 % for 271-360’ time frame) to preclude generation of a pooled effect estimate. Further SWIFT-EVT results on mortality are provided in the Supplement.

Three studies^16,18,19^ reported level of occlusion in relation to time-savings. We performed a sensitivity analysis to determine whether results would be altered by including only these studies that reported on level of occlusion. Heterogeneity remained acceptable (27.45 %) and the overall estimate was that each hour saved was associated with increased survival OR of 1.35 (95 % CI 1.14-1.33). Further results and forest plot provided in the Supplement.

### Translation of meta-analysis pooled effect estimates to absolute measures

**Fig. 4.**
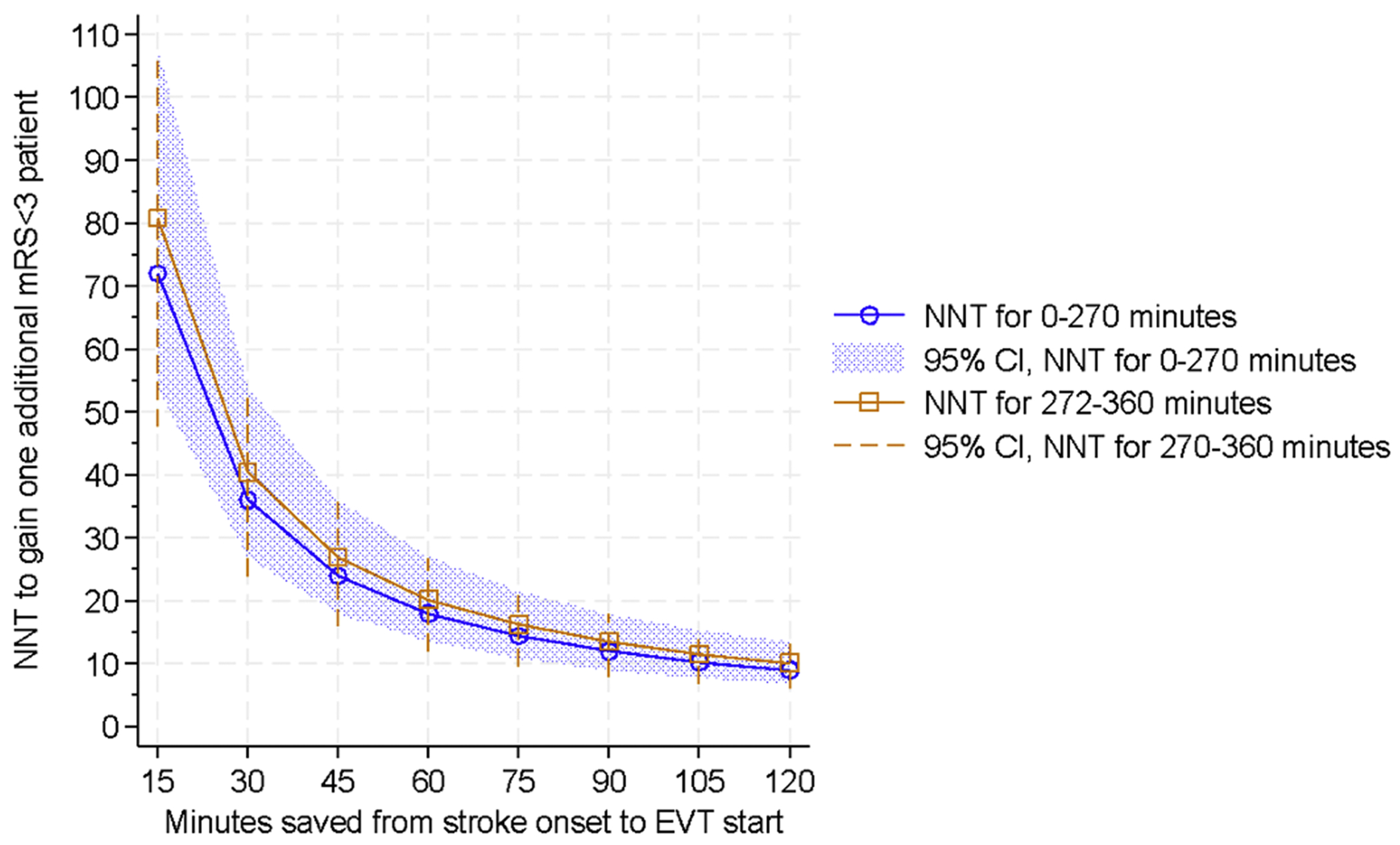
Number needed to treat with varying degrees of pre-EVT time savings, to achieve one additional patient with functional independence (mRS 0-2). NNT – number needed to treat; CI – confidence interval; mRS modified Rankin Scale; EVT – endovascular therapy.

Due to the high-frequency occurrence of the outcome of interest (for both the primary and secondary outcomes), the ORs discussed in SWIFT-EVT overestimate (and thus cannot be taken to approximate) risk ratios. The ORs were instead used as a basis for generating estimates for NNT. Fig. 4 shows sample results for the primary endpoint of functional independence (mRS 0-2), using a baseline rate of half of patients (50 %) expected to achieve mRS 0-2 endpoint at 90 days.

The NNT changes only moderately if the presumed baseline rate of achieving functional independence changes. Changing the baseline functional independence rate from half of cases to a third of cases increases the NNT from 18.0 (the NNT with 50 %) to 21.1; changing the baseline expected to two-thirds of cases (66.7 %) moves the NNT from 18 (at 50 %) to 19.5.

While there was fewer data available for the secondary endpoint (mRS 0-1), the NNT results were broadly similar to those identified for the primary endpoint. With a presumed 1/3^rd^ of cases having expected excellent outcome (mRS 0-1), application of the SWIFT-EVT pooled estimate for this endpoint translates to a NNT of 16.8 (95 % CI 11.9-26.8) for the 0-270’ epoch; the same one-hour time savings in the 271-360’ epoch has a higher (and less precise) NNT of 25.6 (95 % CI 14.9-153.9).

## Discussion

Quantifying the benefit of faster time to EVT is of critical importance for design and improvement of stroke systems of care. Many patients who experience stroke are not near EVT-capable hospitals – over a third of the US population has EMS transport time of greater than one hour to a comprehensive or EVT-capable stroke center.^33^ This has contributed to disparities in access to thrombectomy, particularly for patients in lower income and rural communities.^34^

Our systematic review and meta-analysis estimated that within the initial six hours, for each twenty cases of 60 min reduction in time from LVO AIS symptom onset to EVT start time, there was one additional case of functional independence. Importantly, our findings were based on calculations using a denominator of all EVT cases, so SWIFT-EVT estimations can be broadly useful in application to triage and transfer decisions that are necessarily made without full (or fore-) knowledge of variables such as advanced imaging results or reperfusion success. While not definitive, SWIFT-EVT’s calculations may be useful for system-wide organization and planning of medical care, as well for those making individual patient triage decisions^35^.

We evaluated the time frame up to six hours post-onset. This time frame is consistent with high-grade recommendations for EVT application in LVO AIS. Trials such as DAWN and DEFUSE have demonstrated EVT utility up to 24 h post-onset, but EVT utility in this time frame may be less driven by time issues and more dependent on the presence of robust collaterals resulting in slower progression of irreversible neuronal injury.^36,37^ The six-hour mark from AIS onset has been described as a transition point from a “time is brain” paradigm to one in which time remains important but there is a shift to the mantra “mismatch is brain.”^19^

Recent research has focused on specific populations (e.g. late presenters or more severe stroke^25,38–41^), need for thrombolysis^42^ mechanisms to speed EVT^43^ rather than evaluate its time-criticality, or hospital-centered time frames (*e.g.* door to puncture^44,45^). Although such endpoints are important they do not bear directly on the transport decision-making time frame we aimed to assess and were therefore not included in our analysis.

We assessed time from OTG because this is the time frame most amenable to transport systems’ intervention. Although “door-to-groin puncture” times are important when evaluating performance measures, this was not an endpoint in our analysis. Similarly, time frames that end with actual reperfusion were not included in our analysis because these are affected by a variety of patient factors (e.g. vascular anatomy, thrombus characteristics) and operator factors (e.g. experience) that are associated with both time and also with functional outcome. As noted by others,^46^ the OTG is associated with pre-EVT speed and efficiency and is thus a valued practical and useful clinical marker of performance in all EVT cases (including those in which reperfusion is not achieved).

Symptomatic intracranial hemorrhage (sICH) was also not a primary endpoint for our study. Nogueira^19^ and Mulder^18^ reported association between sICH occurrence and extended treatment window and late start to EVT, respectively. Jahan^17^ reported association between faster onset to puncture and absolute sICH decrease while Cappellari^15^ and Froehler^16^ did not address sICH.

Our calculations are consistent with findings from other studies. The HERMES 2016 meta-analysis of five trials accruing cases through 2014 found that each hour’s delay from onset to EVT resulted in 3.4 % decreased probability of functional independence; for the subset of cases with successful reperfusion each hour’s delay decreased probability of functional independence by 5.2 %.^3^

HERMES results built on a 2013 multicenter study^47^ that assessed the timing cutoff of “successful reperfusion” from either thrombolysis or EVT. The 2013 study reported that each 30 min increase in time from stroke onset to large (ICA or MCA) artery reperfusion worsened mortality (OR 1.21, 95 % CI 1.09-1.34) and was also associated with diminished odds of favorable (0-2) 90-day mRS (OR 0.78, 95 % CI 0.71-0.86). Similarly, SWIFT-EVT has reported that each additional hour from stroke onset to EVT time results in decreased functional independence (mRS 0-2) and varying rates of mortality (*I*^2^ 82 % OR 1.2; 95 % CI 1.1-1.3 for 0-270’ time frame, *I*^2^ 85 % OR 1.1; 95 % CI 0.9-1.1 for 271-360’ time frame). Overall benefits of time savings with thrombolysis were framed differently by an Australian group^48^ that calculated each 15 min time savings in reperfusion provided an equivalent of one month of additional disability-free life. Recent investigations of EVT have reported as clinically significant, pre-EVT time savings of as little as 11 min.^49^ While we only assessed anterior circulation AIS, others have found that faster EVT is also related to improved outcome in other types of ischemia. Joundi, for instance, reported that EVT within six hours of basilar artery occlusion achieves significantly better outcomes than EVT delayed beyond six hours.^50^

The current work is intended to provide a broad frame of reference for those considering either patient- or system-based benefits of time savings for EVT. Fig. 4 demonstrates two points. First, the 95 % CIs for the 0-270’ (shaded region) and the 271-360’ epochs (dashed vertical lines) are sufficiently similar as to question the practical benefits of considering – at least for broad purposes of triage and systems planning – the two time frames separately. Second, the functional independence NNT estimates of just under 20 for a one-hour time savings are nearly identical to the 5.3-5.5 % ARRs calculated in the only two studies (Froehler^16^ and Mulder^18^) reporting such ARRs over SWIFT-EVT’s six-hour window.

In an individual patient, detailed information regarding stroke size or presence of LVO is typically not available at the time of transport decision-making. While novel technology such as VIPS device^51^ and MindRhythm Inc. may offer further patient insight, it remains not widely available in the current or foreseeable future in prehospital setting. For systems, a population-based estimate is preferable for logistics planning on a large scale. In order to optimize the utility of our calculations in the “average” stroke patient, we restricted our analyses to EVT studies that accrued cases across the severity spectrum excluding extreme cases indicated by scores such as ASPECTS.

As hospital-based systems are moving with increased efficiency to expedite stroke care, attention to streamlining care has shifted to the prehospital and transport time frame. For primary ambulance response cases, mobile stroke units have been shown to speed time to thrombolysis in studies that have also suggested time savings to EVT.^52,53^ In interfacility transports, focus on “door-in-door-out” time (*i.e.* time spent at a non-EVT center prior to transfer out for EVT) has suggested improved outcome with shorter patient times at the referring centers.^54, 55^ The individual components of pre-EVT time are important to understand and improve. The evidentiary contribution of SWIFT-EVT is to summarize five recent randomized control trials to demonstrate the clinical outcomes gained by overall streamlining of times to EVT.

One strength of this study is the large number of patients which allows for high precision. In addition, we focused on all patients undergoing EVT renders our result potentially more useful for system-wide planning. Further research is required to understand the time-savings benefit of EVT after six hours of symptom onset.

A limitation of our study is that it included the use of only mRS 0-2 and mRS 0-1 as endpoints to evaluate EVT benefit. However, these clinical variables were selected as *a priori* endpoints as we believe they demonstrate a comprehensive overview of patient independence and outcome post-EVT. Other endpoints such as mRS shift, discharge NIHSS scores, and MoCA scores could equally be examined to determine EVT efficiency and to evaluate various time frames for EVT and recovery optimization.

In conclusion, the SWIFT-EVT calculations offer stroke system planners an informed estimate of one additional functionally independent patient for each 20 cases of one-hour savings pre-EVT. The SWIFT-EVT calculation can be applied to contribute potentially useful patient-centered information to decision-making.

## Conclusions

There was a 22-25 % increase in the odds of achieving functional independence with each hour saved from onset to EVT. While other systematic reviews have similarly analyzed previous onset to EVT time effects on patients, SWIFT-EVT provides a more recent and thorough analysis evaluating good and excellent functional independence. Quantifying this substantial time-dependent benefit, relevant during the entire first six hours post-AIS onset, may be useful to inform patient-specific and systems planning decisions. Further studies should evaluate alternative endpoints such as those mentioned in the discussion to ensure patient treatment optimization and prompt patient selection for EVT.

## Supplementary Material

supplementary material

Supplementary material associated with this article can be found, in the online version, at doi:10.1016/j.jstrokecerebrovasdis.2024.107964.

## Figures and Tables

**Table 1:** Study characteristics.

First author, geography (registry if applicable), publication year	*N* and case accrual time frame	Stroke onset defined based on actual symptom onset or when last seen normal	Key findings on time savings and adjusted survival	Comment
Mulder, The Netherlands (MR CLEAN), 2018^18^	1,488; March 2014 through June 2016	Actual onset 75 %; last seen normal 25 %	Each hour from stroke onset to start of EVT associated with worsening of: 1) Ordinal mRS; OR 0.83 (95 % CI 0.77-0.89) 2) Functional independence (mRS 0-2); OR 0.78 (95 % CI 0.70-0.86) 3) Mortality; OR 0.86 (95 % CI 0.78-0.96)	Linear probability of functional independence outcome over onset-to-EVT window of 90-390’
Froehler et al, Multicenter (STRATIS), 2017	984; August 2014 through June 2016	Last seen well	Increasing time from onset to treatment was significantly associated with lower likelihood of good outcome, with adjusted OR of 0.93 for every additional 30 min delay (95 % CI, 0.89-0.98; *P* = 0.008) When time to treatment accounted for, no additional effect of direct presentation versus transfer on functional outcome (adjusted OR, 1.05; 95 % CI, 0.76-1.45) Absolute rate of functional independence decreased by 5.5 % per hour from alarm to puncture	The accelerated reduction in chance of good outcome after 3.5 h were not observed in this study
Jahan, USA (GWTG), 2019	6,756; January 2015 through December 2016	Actual onset 100 %	Each two hour from stroke onset to start of EVT is associated with worsening of: 1) functional independence (mRS 0-2); OR 3.45 (95 % CI 2.37-5.02), OR 1.69 (95 % CI 1.32 - 2.17), OR 1.39 (95 % CI 1.09-1.79) for 0-2 vs 6-8 h, 2-4 vs 6-8 h, and 4-6 vs 6-8, respectively. 2) sICH; OR 0.35 (95 % CI 0.19-0.67), OR 0.69 (95 % CI 0.51-0.92), OR 0.81 (95 % CI 0.59-1.10) for 0-2 vs 6-8 h, 2-4 vs 6-8 h, and 4-6 vs 6-8, respectively. 3) Mortality; OR 0.51 (95 % CI 0.34-0.75), OR 0.92 (95 % CI 0.75-1.12), OR 1.05 (95 % CI 0.87-1.28) for 0-2 vs 6-8 h, 2-4 vs 6-8 h, and 4-6 vs 6-8, respectively.	Spline at 270 min, splitting time-benefit relationship into two different linear relationships; 0-4.5 and 4.5-8 h.
Cappellari, Italy (Triveneto Registry), 2020	512, September 2018 through December 2018	Actual onset 100 %	1) Door-to-needle time ≤ 60 min (OR 4.005, 95 % CI 1.232–13.016; *p* = 0.021), shorter door-to-groin puncture time (OR 0.991, 95 % CI 0.983–0.999; *p* = 0.023), shorter needle-to-groin puncture time (OR 0.986, 95 % CI 0.975–0.997; p = 0.012), shorter onset-to-groin puncture time (OR 0.994, 95 % CI 0.988–1.000; p = 0.046) reported an association with 3-month excellent outcomes (mRS 0-1). 2) Shorter door-to-groin puncture time (OR 0.991, 95 % CI 0.984–0.998; p = 0.009), door-to-groin puncture time ≤ 90 min (OR 12.146, 95 % CI 2.193–67.280; p = 0.004), shorter needle-to-groin puncture time (OR 0.983, 95 % CI 0.972–0.995; *p* = 0.004), and shorter onset-to-groin puncture time (OR 0.993, 95 % CI 0.987–0.999; *p* = 0.014) reported an association with 3-month favorable outcomes (mRS 0-2).	Multiple critical times such as door-to-needle, needle-to-groin, onset-to-needle, door-to-groin, and onset-to-groin time for thrombectomy have been found to influence stroke outcome.
Nogueira, International Multicenter (Trevo Retriever Registry), 2018	1603; November 2013 through May 2017	Last seen well	Stroke onset to EVT time in patients treated early (<6h) was significantly associated with overall degree of functional disability in the ordinal mRS distribution (aOR for >1-point mRS shift: 0.75; 95 % CI [0.66-0.86], P <0.001) and good outcomes (aOR for mRS 0-2: 0.73; 95 % CI [0.62-0.86], *P* < 0.001) at 90 days. No correlation between stroke onset to EVT time and overall degree of functional disability (aOR for >1-point mRS shift: 0.96; 95 % CI [0.90-1.02], *P* = 0.15) or good outcomes (aOR for mRS 0-2: 0.97; 95 % CI [0.90-1.04], *P* = 0.41) at 90 days in patients treated within the extended time window (>6-24 h). Overall cohort time to treatment outcomes across the early (aOR for >1-point mRS shift: 0.80; 95 % CI [0.72-0.89], *p* < 0.001: aOR for mRS 0-2: 0.79; 95 % CI [0.69-0.90], *P* = 0.001) and extended (aOR for >1-point mRS shift: 0.96; 95 % CI [0.91-1.004], *P* = 0.071; aOR for mRS 0-2:0.98; 95 % CI [0.92-1.05], *P* = 0.554) time windows.	Findings in this study highlight the importance of “time is brain” concept in the early window. This study reinforces the importance between TLSW to puncture and functional independence at 90 days (n=1173; OR for 90-day mRS 0-2: 0.79; 95 % CI [0.69-0.90] per 1-h delay) with the added advantage of nearly doubled sample size.

## Data Availability

The datasets used and/or analyzed during the current study are available from the corresponding author on reasonable request.
