# Supplementary material for: Success with incrementally faster times to endovascular therapy (SWIFT-EVT): A systematic review and meta-analysis
Source: J Stroke Cerebrovasc Dis. Author manuscript; Available in PMC 2026 Mar 28. (PMC13032828; doi:10.1016/j.jstrokecerebrovasdis.2024.107964)
Supplement: supplementary material [file NIHMS2155539-supplement-supplementary_material.docx]

**Supplementary Material**

**S1: PRISMA checklist, search strategy, and results screening**

**S2: Details on studies comprising the review set**

**S3: Extended results: meta-analysis**

**S4: GRADE and RoB2 tables**

**S1: Search strategy and screening of results**

*PRISMA checklist*

| **Section** | **Checklist item** |
| --- | --- |
| *Title* | 1. Identify the report as a systematic review |
| *Materials & Methods* | 2. Provide an explicit statement of the main objective(s) or question(s) the review addresses |
| *Materials & Methods* | 3. Specify the inclusion and exclusion criteria for the review |
| *Supplement* | 4. Specify the information sources (such as databases, registers) used to identify studies and the date when each was searched |
| *Supplement* | 5. Specify the methods used to assess risk of bias in the included studies |
| *Materials & Methods; Supplement* | 6. Specify the methods used to present and synthesize results |
| *Results and Supplement* | 7. Give the total number of included studies and participants and summarize relevant characteristics of studies |
| *Results and Supplement* | 8. Present results for main outcomes, preferably indicating the number of included studies and participants for each. If meta-analysis was done, report the summary estimate and confidence/credible interval. |
| *Discussion and Supplement* | 9. Provide a brief summary of the limitations of the evidence included in the review (such as study risk of bias, inconsistency, and imprecision) |
| *Discussion* | 10. Provide a general interpretation of the results and important implications |
| *Title page* | 11. Specify the primary source of funding for the review |
| *Methods* | 12. Provide the register name and registration number |

*Search strategy: search terms*

We used a similar search strategy to that reported in a recent (2023) meta-analysis of AIS.^60^ The search was limited to humans, and included both peer-reviewed publications and gray literature (*e.g.* dissertations, conference proceedings). The search was executed using the Harvard On-Line Library Information System (HOLLIS) on December 1 2023, and repeated on 1 January 2024. Search terms were: PubMed, Scopus, CENTRAL, Google Scholar, ClinicalTrials.gov. The following keywords and their related MeSH terms were used: (Endovascular OR Thrombectomy OR Embolectomy OR Aspiration Thrombectomy OR Percutaneous Aspiration Thrombectomy) AND (Stroke OR Apoplexy OR CVA OR Cerebrovascular Accident OR Cerebral Stroke OR Cerebrovascular Accident OR Cerebrovascular Apoplexy OR Cerebrovascular Stroke) AND (Clinical Trial).

*Post-HERMES time frame and avoidance of replication of cases from HERMES*

The intent of the current meta-analysis was to complement and extend the work of Saver and colleagues in their 2016 meta-analysis, HERMES.^3^ It was therefore important to eliminate duplication of patients in the current meta-analysis, who had already been included in the previous work.

HERMES assessed studies from five different trials.^4-8^ In each of those trials, studies accrued patients through some part of 2014. Studies that included patients from the same registry, but who were not included in the time frame covered by HERMES, were eligible for inclusion in this analysis.^19^

*Screening of results*

The initial search return of over 14,000 records was reviewed by title and abstract, by two authors (SHT and DWS). Any potentially eligible records were retrieved in full-text version; these were also reviewed by at least two authors (SHT and at least one additional co-author). Approximately 100 records were reviewed in full-text version.

The primary reason for exclusion of records was non-reporting of a “slope” coefficient relating a specific degree of time savings pre-EVT, to a specific increment in an mRS endpoint. Most records that were discarded, assessed the value of time savings in dichotomized categories (*e.g.* “early” or “late” EVT) or reported on group comparisons (*e.g.* primary presentation to stroke center *vs.* interfacility transport) without patient-level analysis of effect estimate of time savings on outcome.

In an effort to maintain broad-based relevance to our pooled effect estimate, we included only studies that assessed time-mRS relationships in “all comers” with anterior LVO AIS. Since stroke systems planning should not be expected to require referring-hospital advanced neuroimaging results, we excluded any studies that were focused only on a subset of stroke (usually by severity).^24,37-39,53-61^

Any articles that were judged questionable for inclusion, were assessed by at least two senior authors (SHT and either JAE, AS, or MS). With the *caveat* that articles often had multiple reasons for exclusion, the figure 1 in the manuscript denotes the article screening and selection process.

**S2: Details on studies comprising the review set**

The studies in the review set included differing endpoints and differing time windows. Notes on each study’s endpoints are provided in this section.

The Froehler study^17^ primarily focused on the STRATIS group’s comparison of direct presenters to the CSC *vs.* patients who were transferred to the CSC. As such, much of the analytic comparison was “direct *vs.* transfer” groups and did not include a specific tie to OTG time. The one endpoint for which OTG time was reported as a direct predictor, was the achievement of 90-day functional independence. This “functional independence” endpoint had a reported OR for each half-hour of OTG savings; the functional independence endpoint was also reported as an absolute risk reduction for each hour of OTG time savings. Other endpoints (*e.g.* mortality) were assessed, but no results were provided for these endpoints as a function of time.

The Mulder study^19^ reported on MR CLEAN data (post-HERMES) and OTG association with mRS as a dichotomous outcome of “good” functional independence (mRS 0-2). OTG association with excellent mRS (0-1) was also reported, as was 90-day mortality. Mulder assessed the study group of “cases undergoing successful reperfusion” but in this group the OTG time was *not* the predictor that was reported; the authors rather reported the overall onset to reperfusion time. The successful-reperfusion results were not included in this analysis.

The Jahan paper^18^ reported detailed results for Get With The Guidelines cases’ OTG and a breadth of endpoints. The mortality endpoint was assessed at hospital discharge, rather than 90 days, but any underestimation of 90-day mortality was mitigated by the hospital discharge “mortality” assessment including patients discharged to hospice.

The Jahan authors point out that their findings were quite similar to those reported by Mulder^19^ and reviewed in this meta-analysis; Jahan’s group points out that the larger study *n* for their cohort was likely responsible for their finding what Mulder and some others have not: a non-linear association between OTG and outcomes.

Cappellari^16^ focused on regional stroke care in Italy, evaluating presentation sites in the Triveneto registry. Cappellari also reported on all three endpoints we assessed: mRS 0-2, mRS 0-1, and mortality. The reported ORs were for a one-minute change; these were translated to 60-minute coefficients for our analysis.

Nogueira,^20^ writing for the Trevo Registry and DAWN groups, was primarily analyzing (and demonstrating effectiveness of) an extended time window for EVT. Limited results on OTG time were available, but a specific coefficient for OTG and functional independence outcome was provided.

All of the studies reported on the primary endpoint of this meta-analysis: mRS of 0-2. Two (Froehler^17^ and Nogueira^20^) did not include reporting on OTG and mRS of 0-1; these two studies also did not report a coefficient for OTG and mortality odds. The other three studies^16,18,19^ reported on all three endpoints of focus for this meta-analysis.

**S3: Extended results: meta-analysis**

*Table of detailed results for meta-analysis for three endpoints*

The detailed results for meta-analysis include two measures of heterogeneity, *I*^2^ and Cochrane’s Q, and also a *p* value assessing likelihood that the overall effect estimate differs from the null value. Summary of each of these is presented in the table below.

| **Endpoint** | **Time frame** | ***I*^2^** | ***p*, Cochrane’s Q** | ***p*, pooled effect estimate departure from null** |
| --- | --- | --- | --- | --- |
| mRS 0-2 | 0-270’ | 40% | .48 | <.01 |
| mRS 0-2 | 271-360’ | 58% | .22 | <.01 |
| mRS 0-1 | 0-270’ | 27% | .28 | <.01 |
| mRS 0-1 | 271-360’ | 60% | .13 | .02 |
| Mortality | 0-270’ | 94% | <.01 | .68 |
| Mortality | 271-360’ | 88% | <.01 | .71 |

**Table S3-1.** Detailed summary of meta-analysis results

*Sensitivity analysis: Sensitivity to model selection*

This section reports results for the DL random effects model. These results were not substantially different from those obtained with the HKSJ model.

The table below also reports results for the fixed-effect model, which are used as a measure of heterogeneity (*i.e.* to assess the degree to which they departed from the random effects model estimates).

Neither the DL estimates nor the fixed-effect estimates showed important departures from the HKSJ model; all estimates were within 10% of HKSJ and all 95% CIs substantially overlapped. The findings support conclusions of lack of results sensitivity to model selection, and also do not indicate heterogeneity.

| **Endpoint** | **Time frame** | ***I*^2^** | **DL model: pooled OR estimate (95% CI)** | **Fixed-effects model: pooled OR estimate (95% CI)** |
| --- | --- | --- | --- | --- |
| mRS 0-2 | 0-270’ | 0% | 1.2 (1.2-1.3) | 1.2 (1.2-1.3) |
| mRS 0-2 | 271-360’ | 30% | 1.2 (1.1-1.3) | 1.2 (1.1-1.3) |
| mRS 0-1 | 0-270’ | 21% | 1.3 (1.2-1.5) | 1.3 (1.2-1.5) |
| mRS 0-1 | 271-360’ | 51% | 1.2 (1.0-1.4) | 1.2 (1.1-1.3) |
| Mortality | 0-270’ | 82% | 1.1 (.93-1.3) | 1.2 (1.1-1.3) |
| Mortality | 271-360’ | 85% | .96 (.78-1.2) | 1.1 (0.9-1.1) |

**Table S3-2.** Pooled effect estimates from alternative (DL) model and fixed-effects model

*Galbraith plots*

Galbraith plots for both the primary and secondary endpoints, for both the earlier and later time frames, indicated neither heterogeneity nor study outliers. Plots are depicted in Figures S3-1 and S3-2.


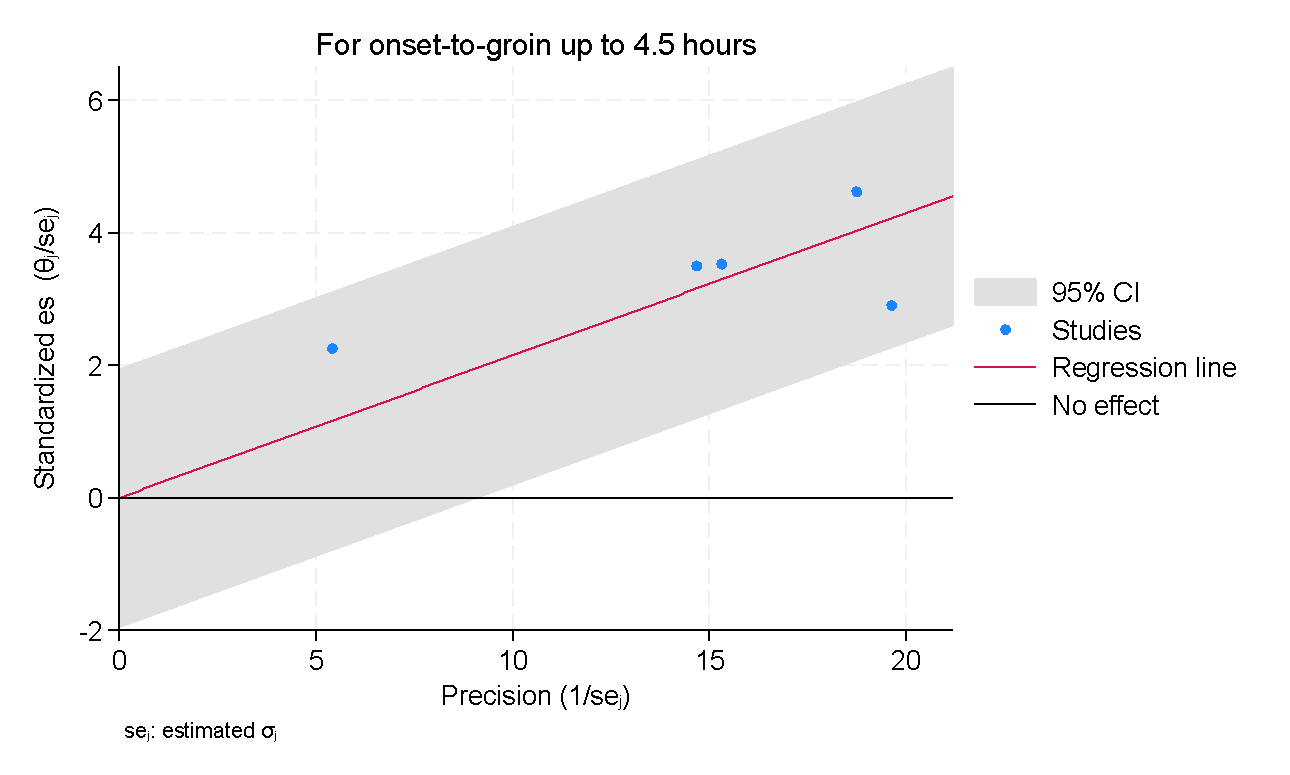


**Figure S3-1a.** Galbraith plots for mRS 0-2: Early (0-270’) time frame


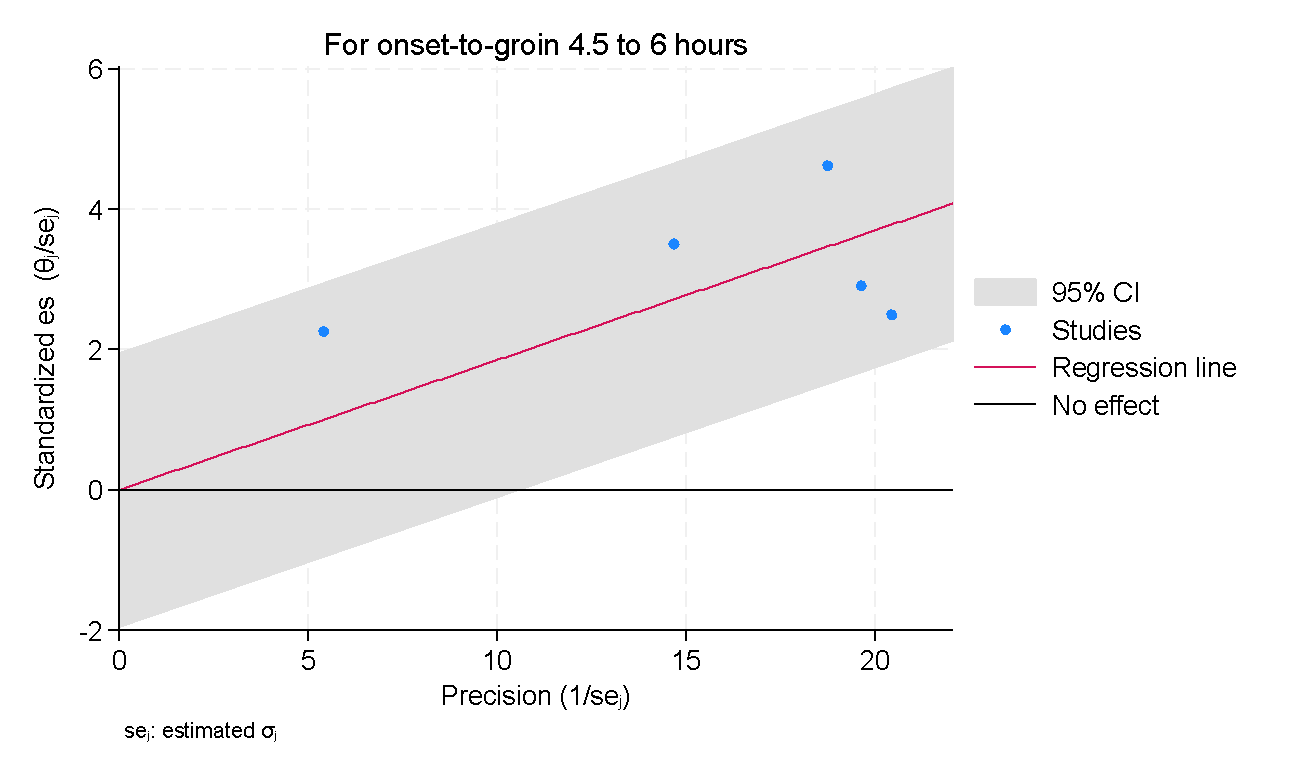


**Figure S3-1b.** Galbraith plots for mRS 0-2: Later (271-360’) time frame

*
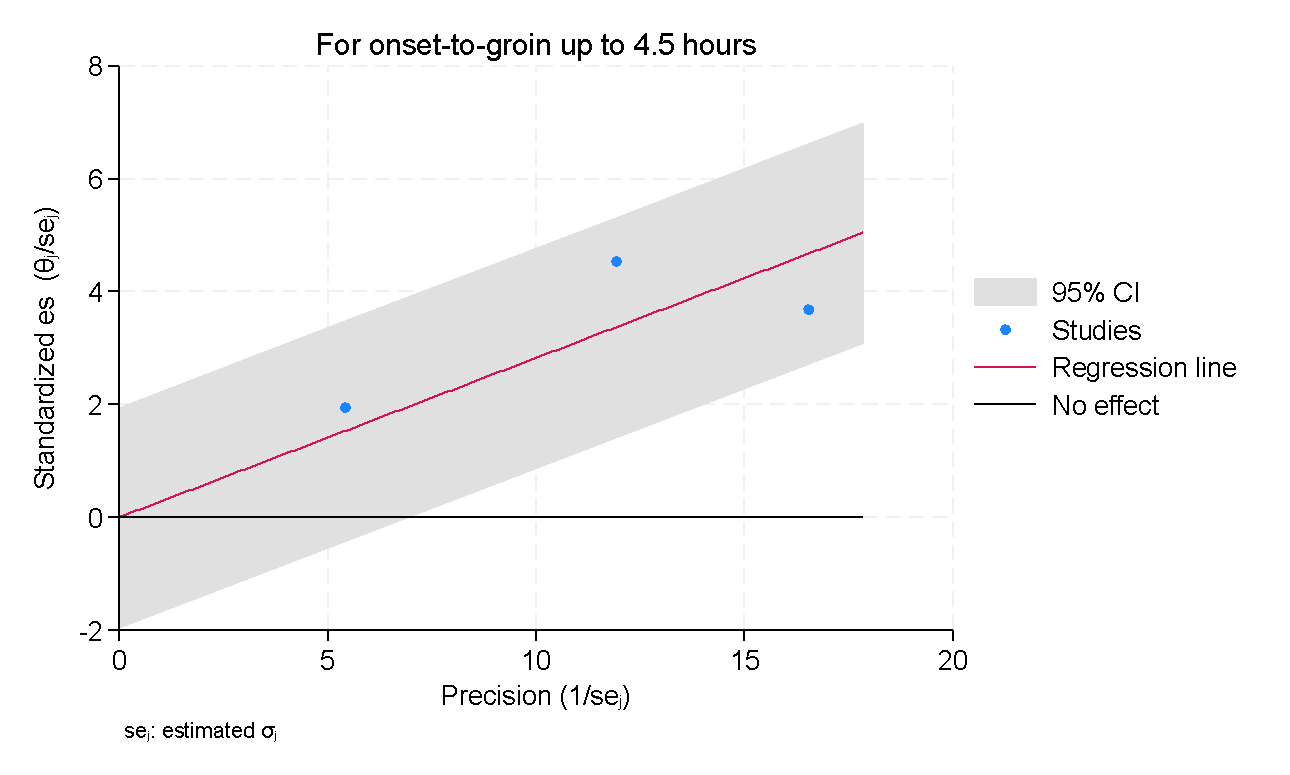
*

**Figure S3-2a.** Galbraith plots for mRS 0-1: Early (0-270’) time frame

*
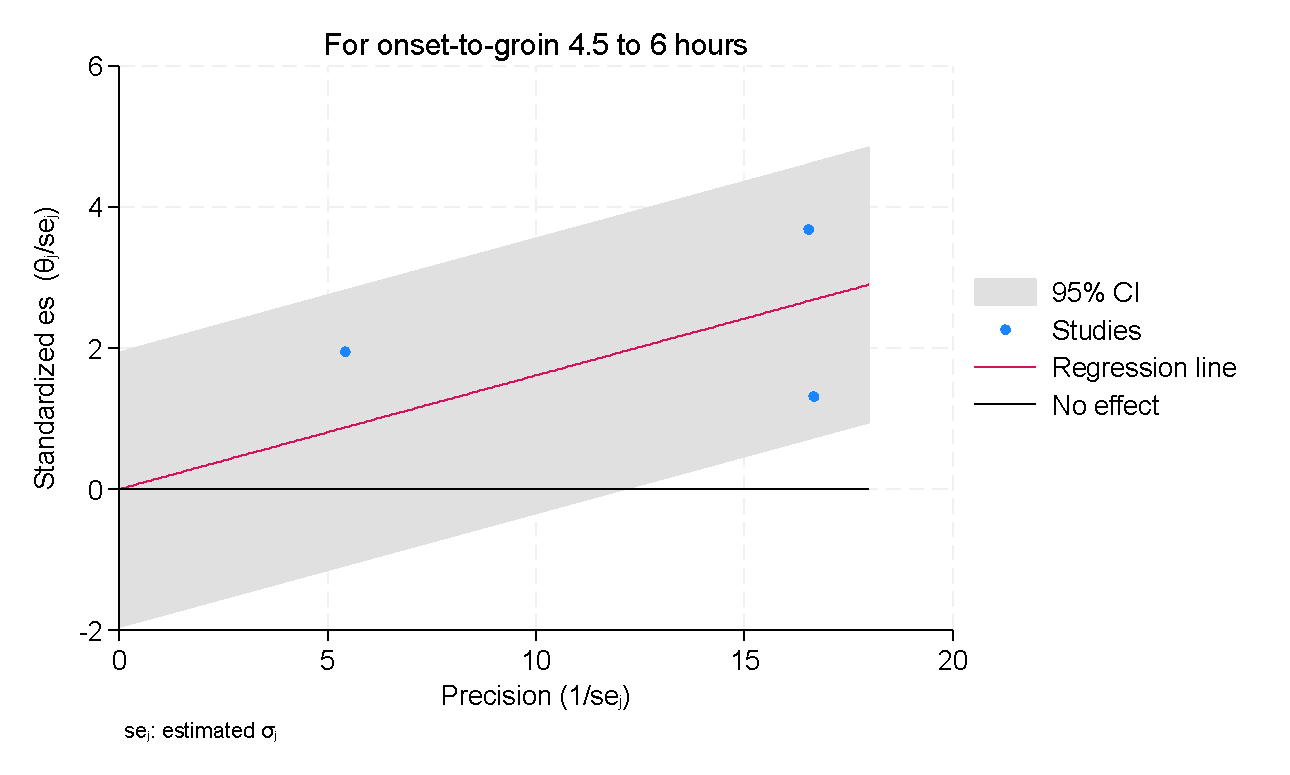
*

**Figure S3-2b.** Galbraith plots for mRS 0-1: Later (271-360’) time frames

*Funnel plots*

The funnel plots for the primary and secondary endpoints are shown in Figures S3-3 and S3-4. There was insufficient study *N* to enable formal funnel asymmetry analysis.^28^


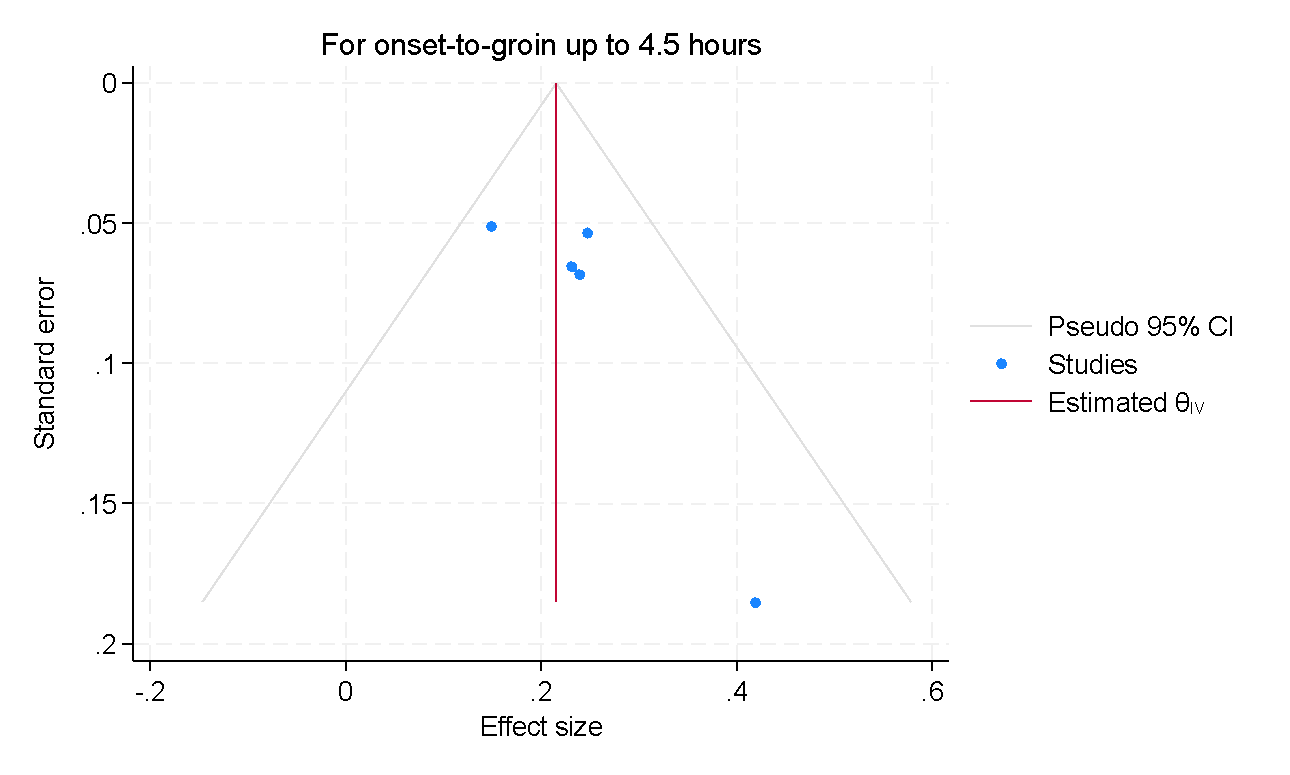


**Figure S3-3a.** Funnel plots for mRS 0-2: Early (0-270’) time frame

**Figure S3-3b.** Funnel plots for mRS 0-2: Later (271-360’) time frame
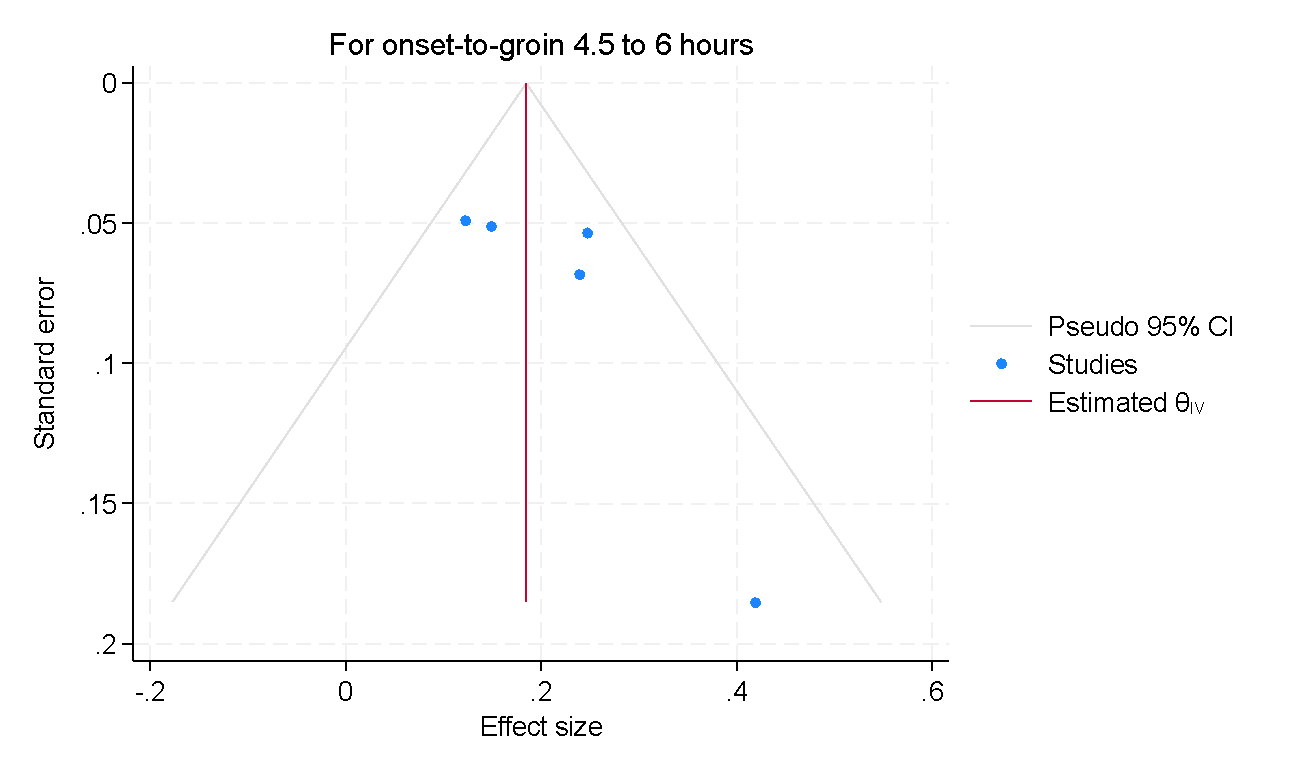


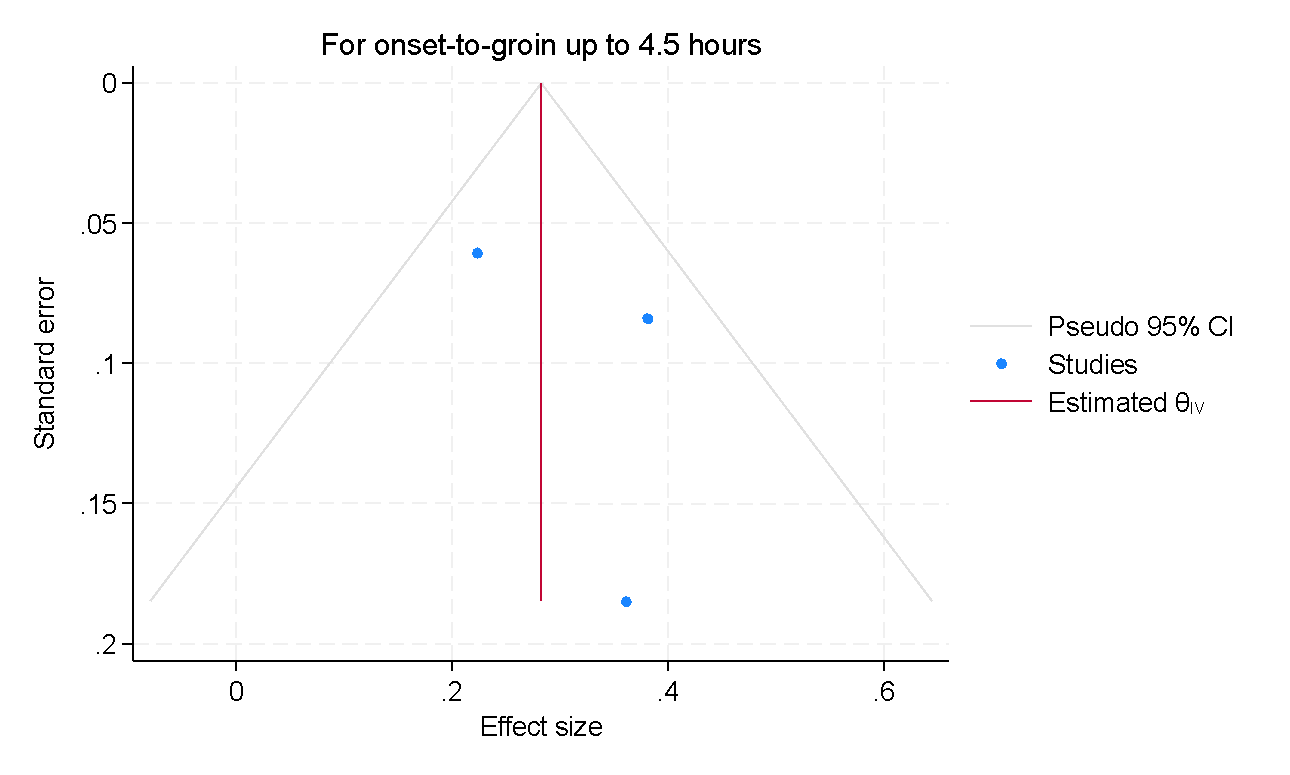


**Figure S3-4a.** Funnel plots for mRS 0-2: Early (0-270’) time frame


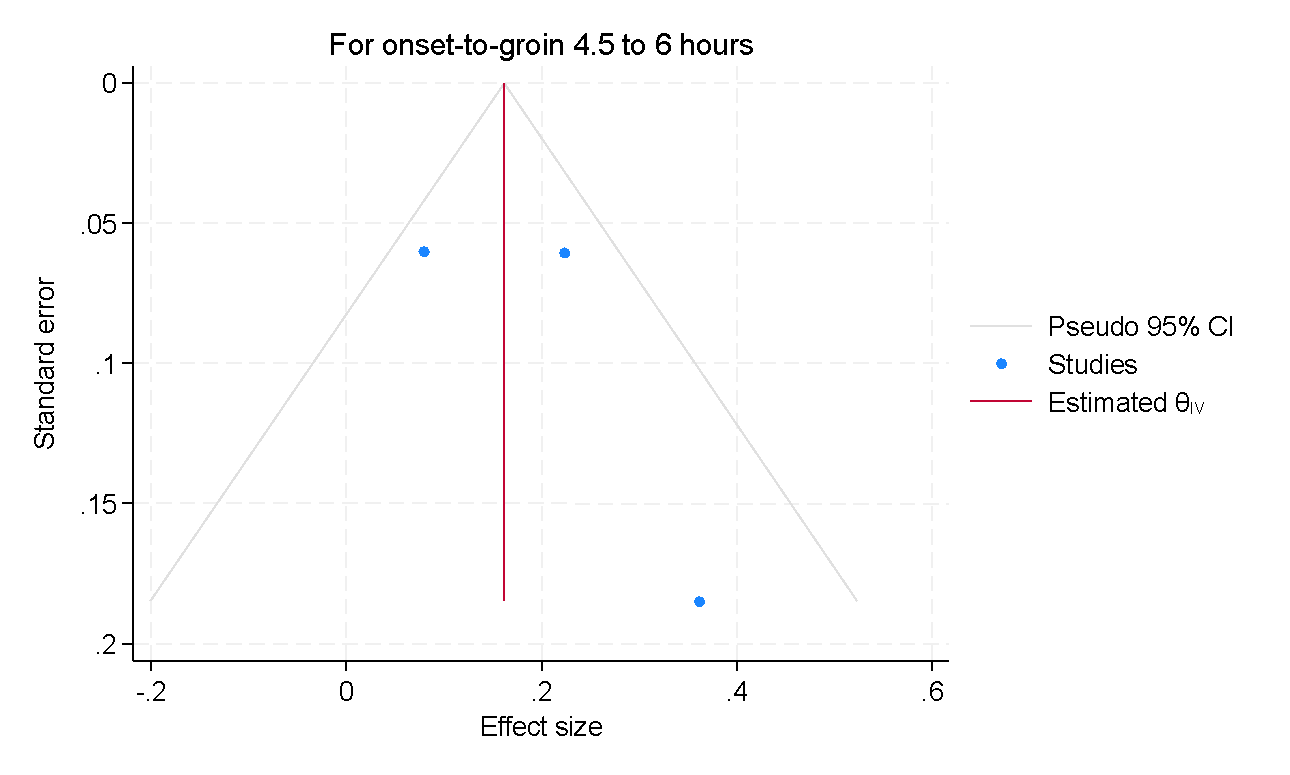


**Figure S3-4b.** Funnel plots for mRS 0-2: Later (271-360’) time frame

*Cumulative meta-analysis*

Cumulative meta-analysis for the primary and secondary endpoints, for both the earlier (0-270’) and later (271-360’) time frames, is presented in this section. Cumulative meta-analysis was organized by increasing study *n*.

For the primary endpoint of mRS 0-2, the results stabilized relatively quickly. Increasing *n* was characterized by a stabilizing effect estimate that remained essentially unchanged over larger studies (Table S3-5).


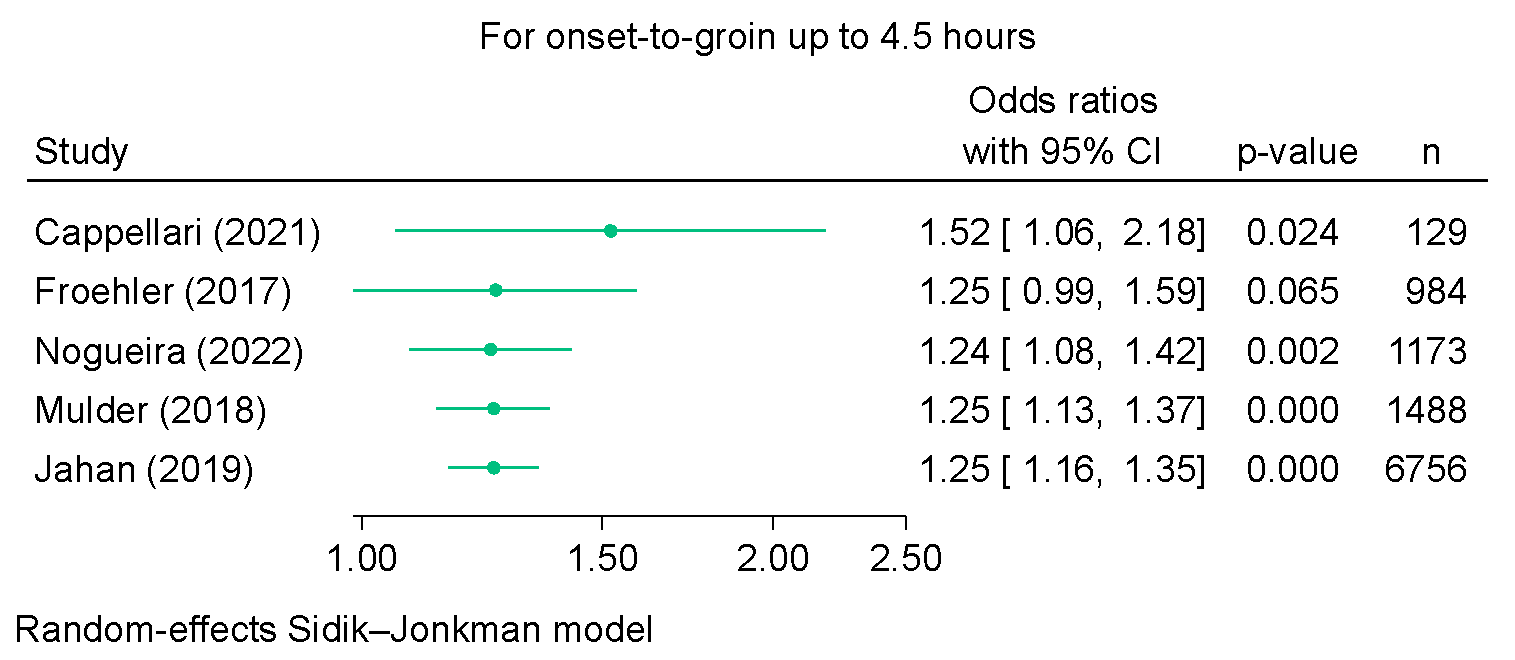


**Figure S3-5a.** Cumulative analysis for mRS 0-2: Early (0-270’) time frame


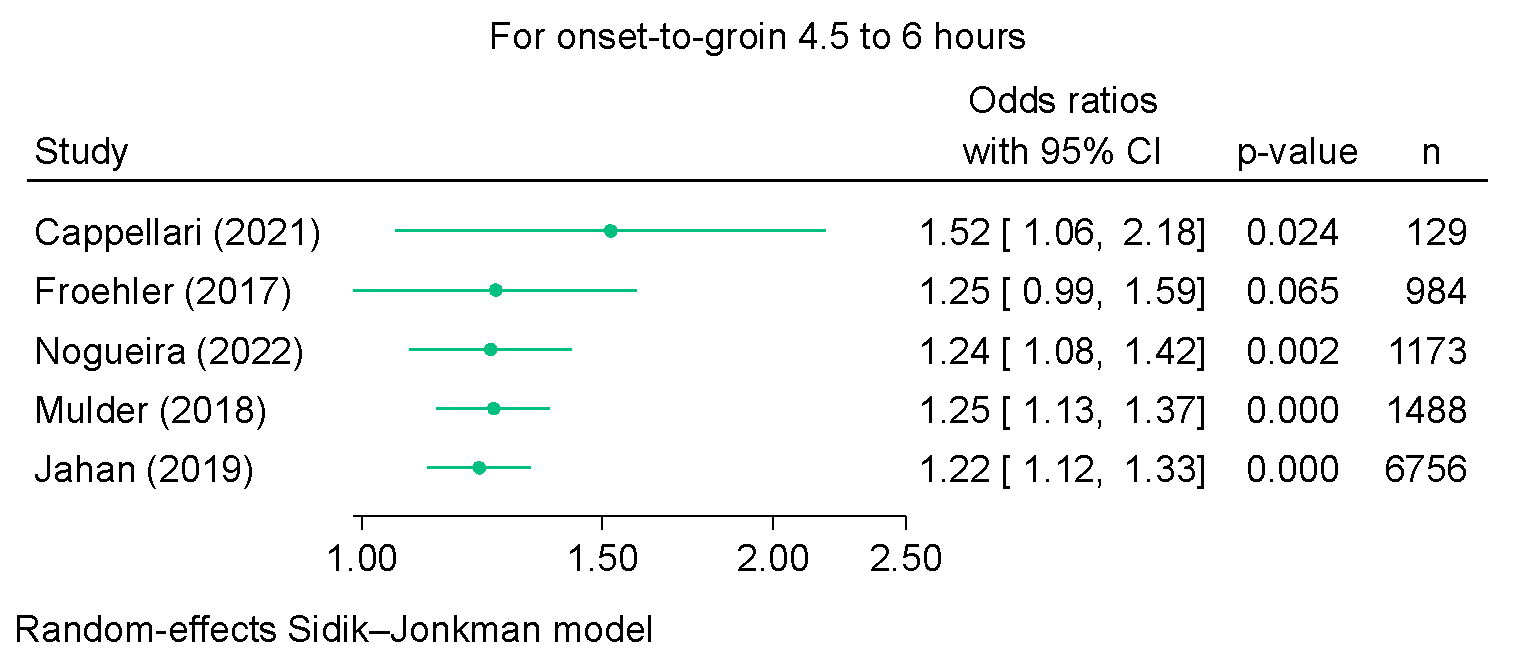


**Figure S3-5b.** Cumulative analysis for mRS 0-2: Later (271-360’) time frame

For the secondary endpoint of mRS 0-1, there was less stability in the meta-analysis results (which came from only *N* = 3 studies rather than *N* = 5 for the primary endpoint). For the early period (0-270’) the effect estimate appeared to become relatively stable as studies with increasing *n* were assessed. For the later period (271-360’), however, increasing study *n* appeared to produce a decreasing effect estimate. Figure S3-6 depicts the results for mRS 0-1.


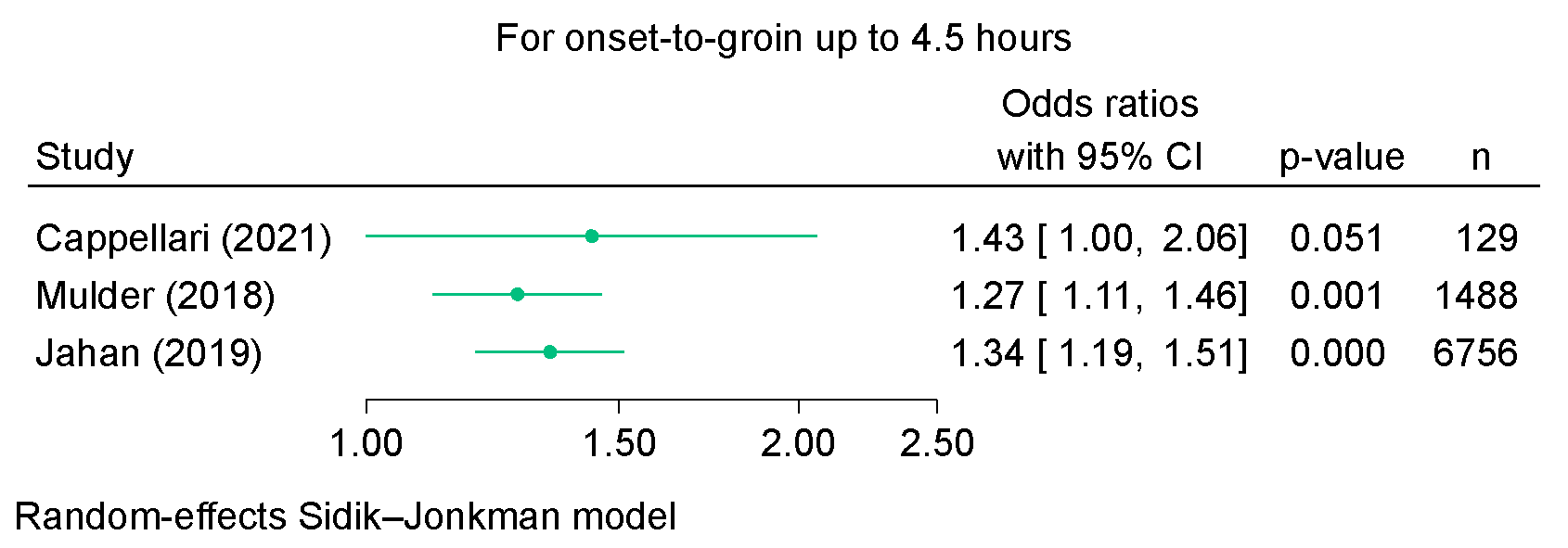


**Figure S3-6a.** Cumulative analysis for mRS 0-2: Early (0-270’) time frame


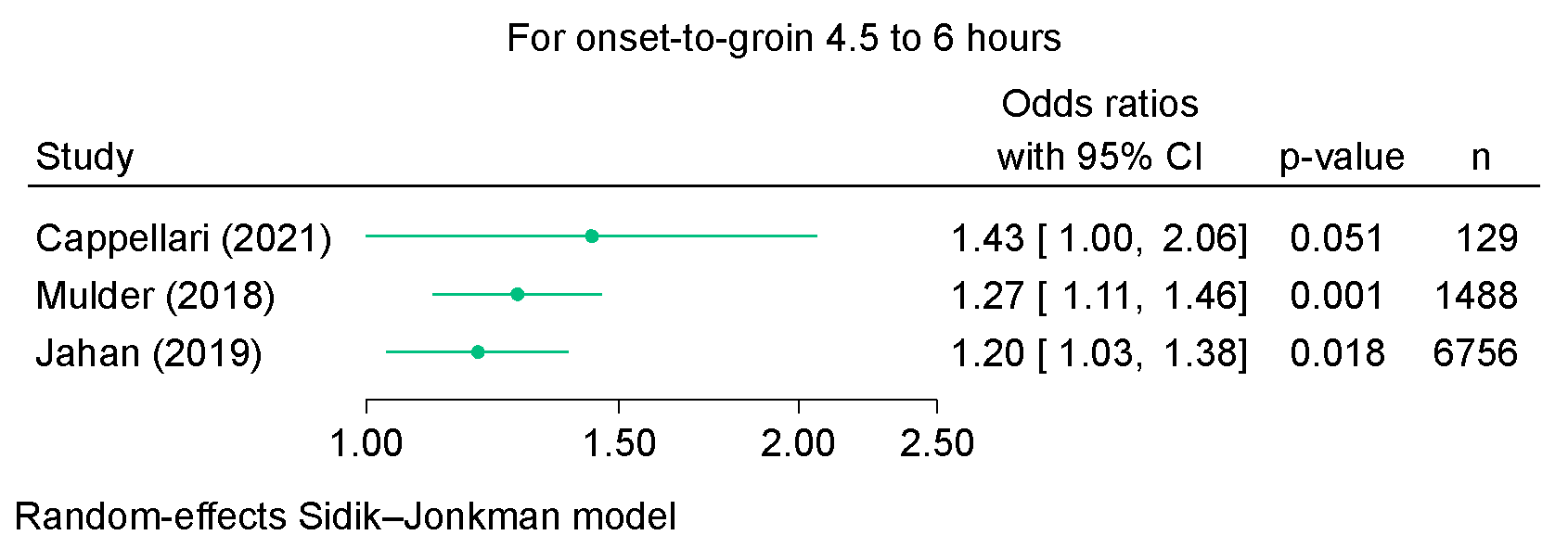


**Figure S3-6b.** Cumulative analysis for mRS 0-2: Later (271-360’) time frame

*Omitted-study meta-analysis*

To ascertain the degree to which individual study results executed inordinate leverage on the overall results, omitted-study (leave-one-out) meta-analysis was executed. The plots in this section show the effect estimate that would be generated if the named study were not included in the calculations.

Omitted-study analysis for the primary endpoint of functional independence suggested that no individual study exerted undue leverage. Results did not change substantially when any one of the five studies was excluded. The results are shown in Figure S3-7.


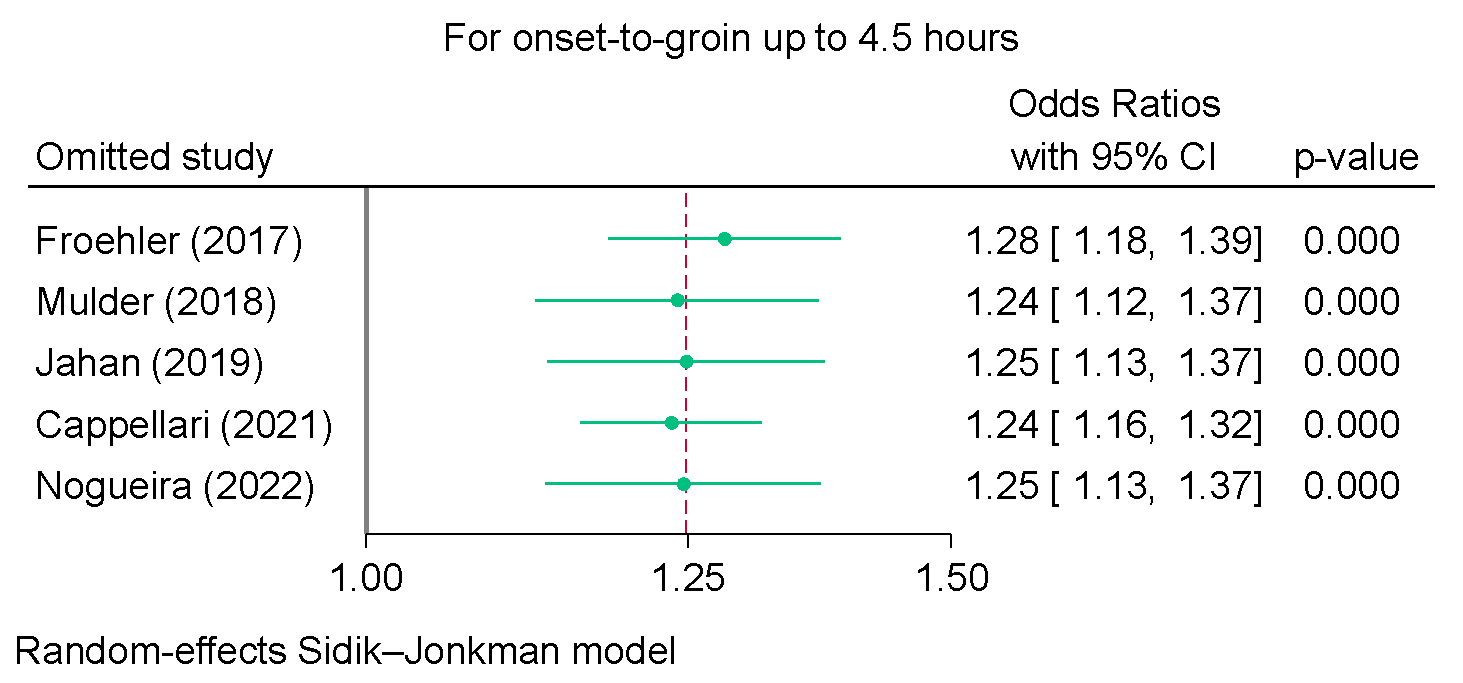


**Figure S3-7a.** Leave-one-out plot for functional independence: Early (0-270’) time frame


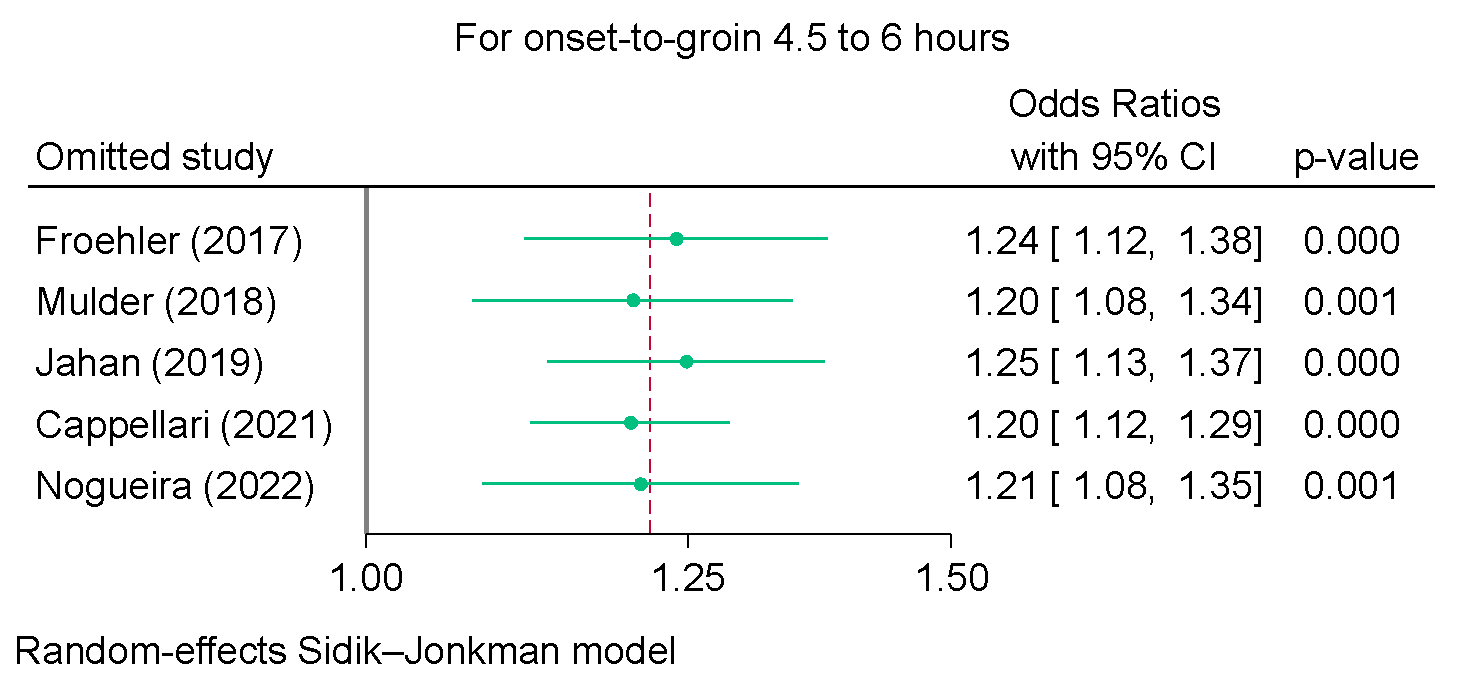


**Figure S3-7b.** Leave-one-out plot for functional independence: Later (271-360’) time frame

Omitted-study analysis for the secondary endpoint of mRS 0-1 (Figure S3-8) also suggested no undue study leverage, although results were less stable than those for the primary endpoint. This is to some degree expected, since with overall *N* = 3 for this endpoint there were only two remaining studies when any one was excluded.


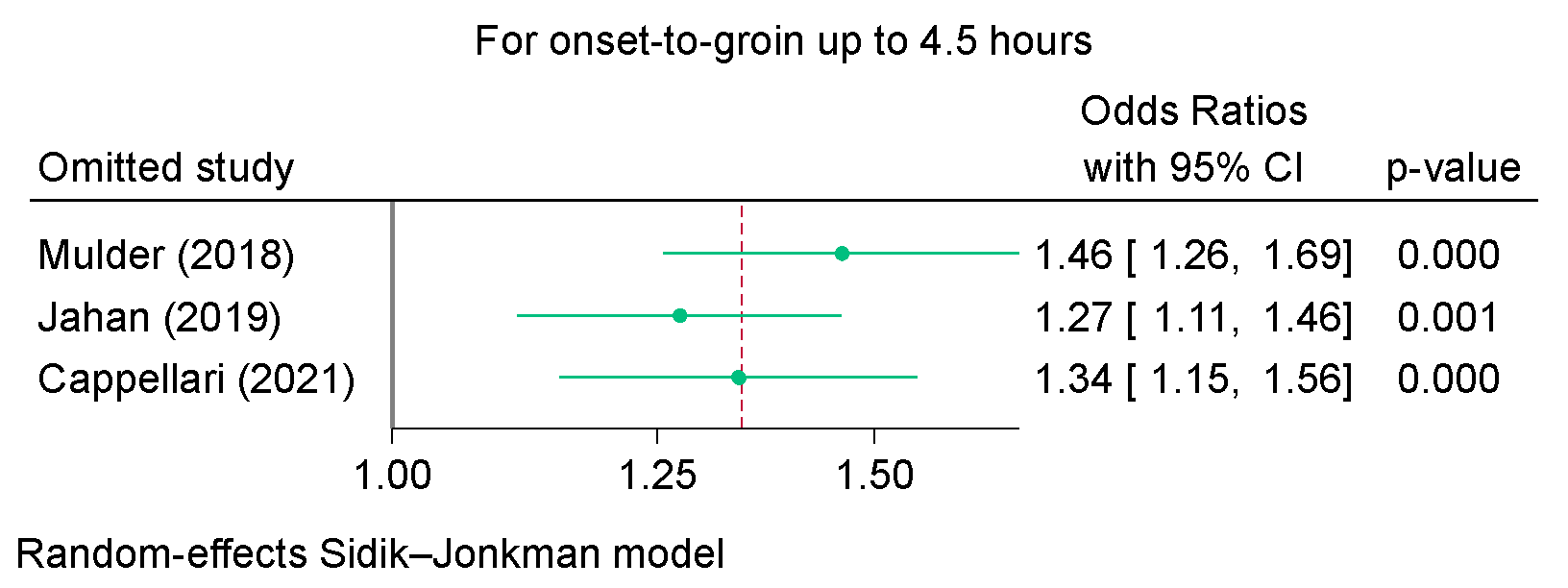


**Figure S3-8a.** Leave-one-out plot for mRS 0-1: Early (0-270’) time frame


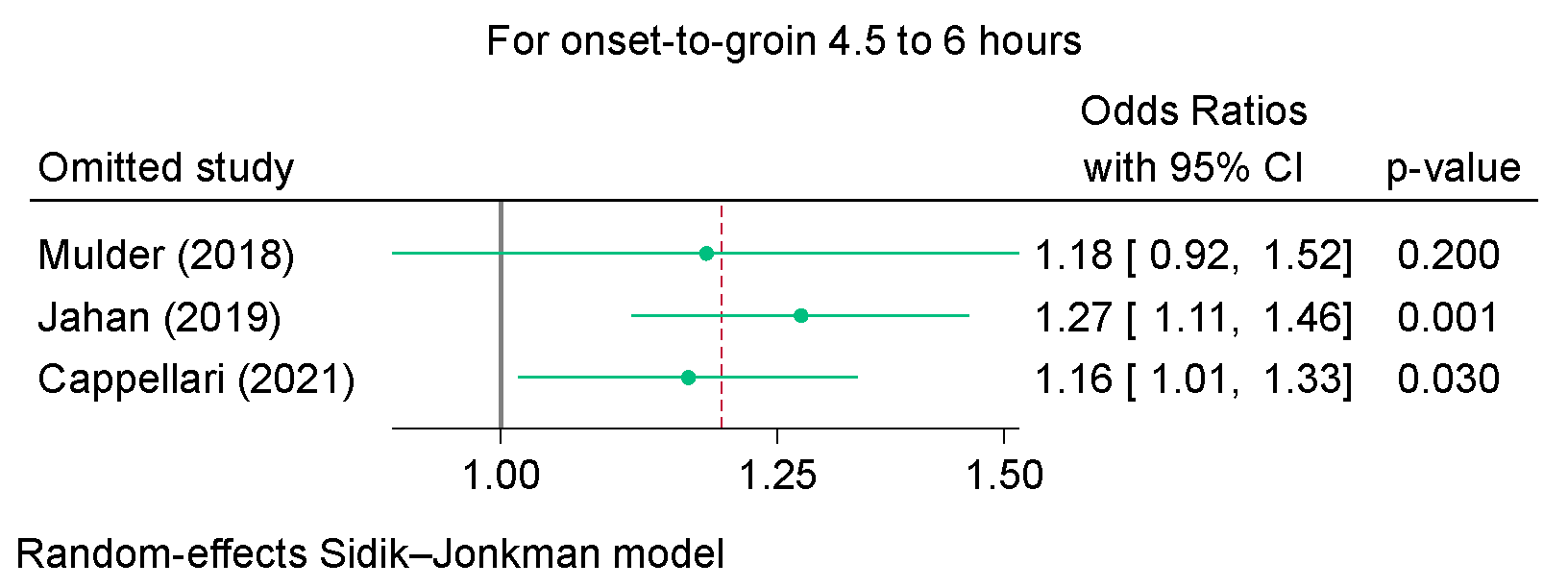


**Figure S3-8b.** Leave-one-out plot for mRS 0-1: Later (271-360’) time frame

*Meta-analysis for mortality endpoint*

While not a primary focus of this study, some studies reported on the effect of time savings on mortality. However, high *I*^2^ for both time frames precluded generation of a pooled effect estimate. Cochrane’s Q assessment for the mortality endpoint also rejected the null hypothesis of homogeneity (*p* <.01 for both the 0-270’ and the 271-360’ time frames).

Unlike the case with either the primary or secondary endpoints, the mortality endpoint meta-analysis did not reject the null hypothesis of no effect of time-savings on the endpoint (survival). This failure to identify an effect of time savings on mortality was noted for both the early (*p* = .68) and the late (*p* = .71) time frames.

Given the heterogeneity of study findings, results for any meta-analysis pooled effect estimate are not presented. A forest plot without pooled effect estimate is shown in Figure S3-9.


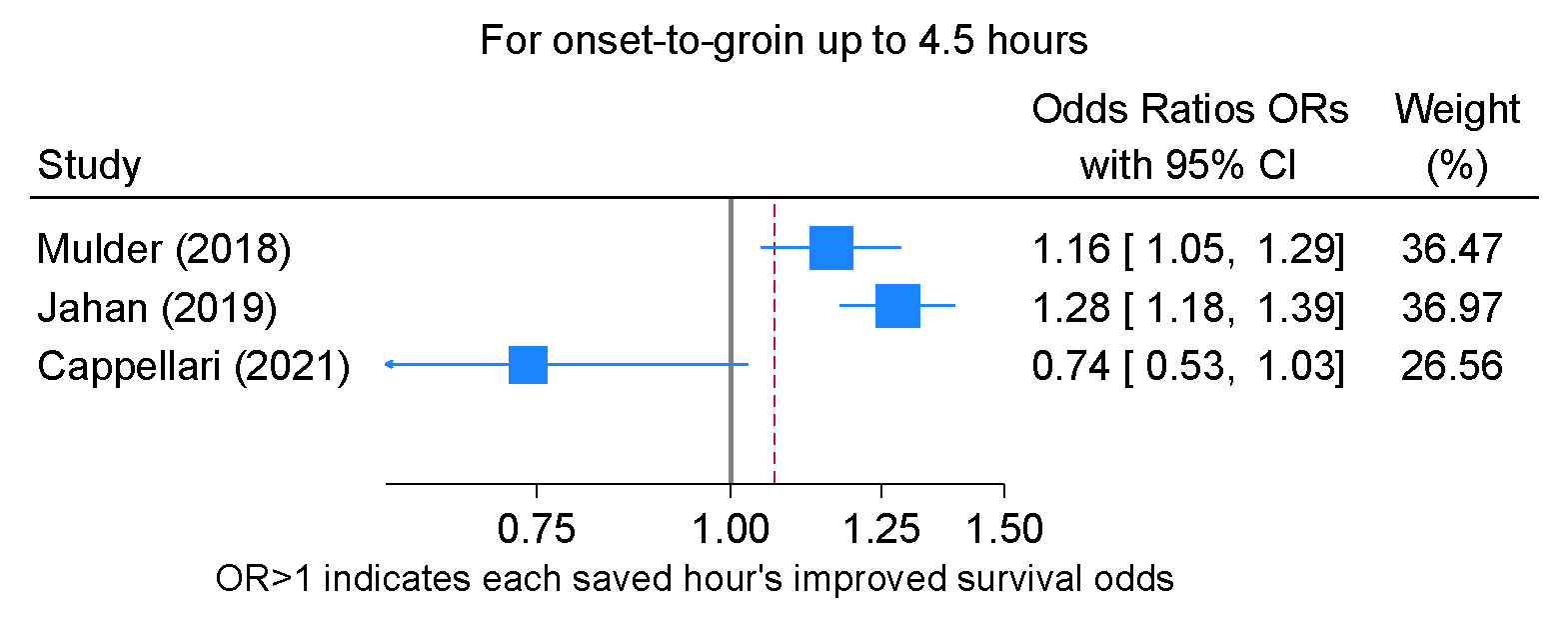


**Figure S3-9a.** Forest plot for primary endpoint of time savings (each hour) and mortality (0-270’ time frame)


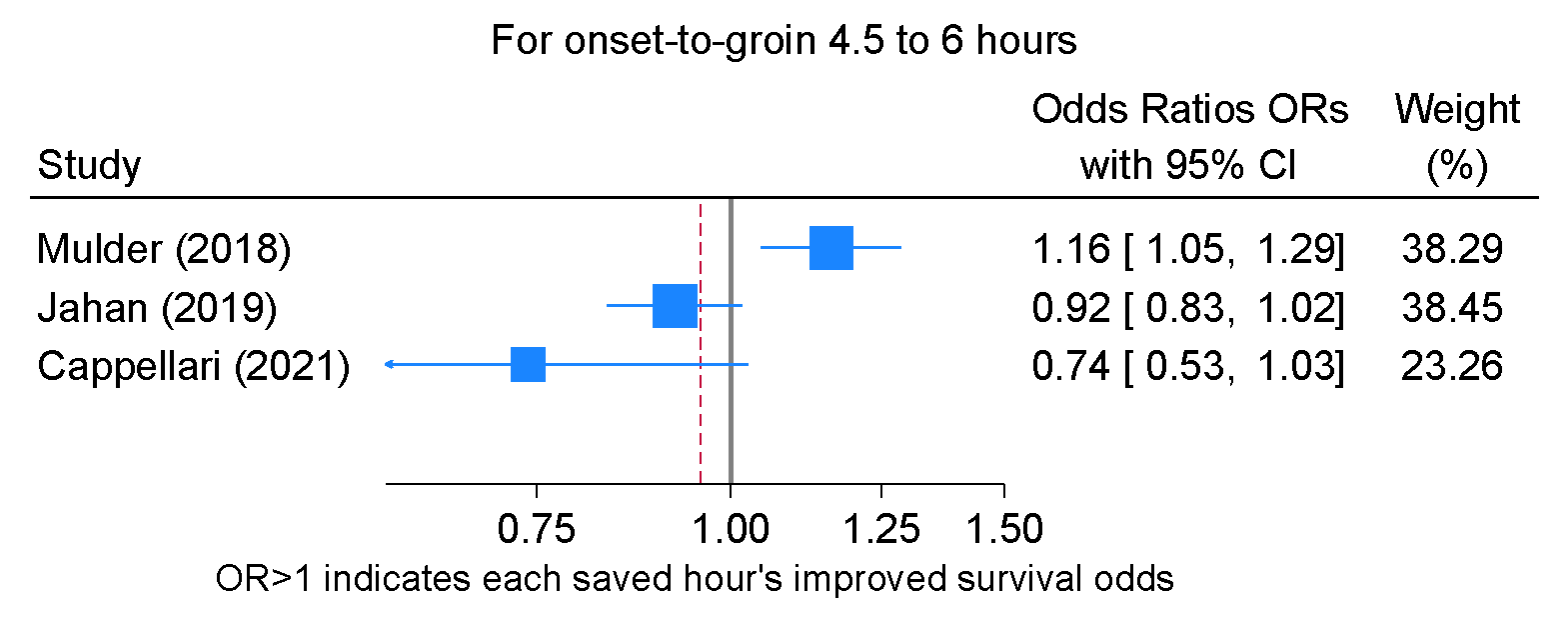


**Figure S3-9b.** Forest plot for primary endpoint of time savings (each hour) and mortality (271-360’ time frame)

*Sub Analysis for Level of Occlusion Endpoint*

We performed a sensitivity analysis to determine whether the results would be altered by including only those studies that reported on the level of occlusion. The sensitivity analysis dataset included studies other than those by Jahan and Capillari. Results (shown below) were generally in line with those found in the main analysis. The heterogeneity remained acceptable (27.45%) and the overall estimate was that each hour saved was associated with increased survival OR of 1.35 (95% CI 1.14-1.33).


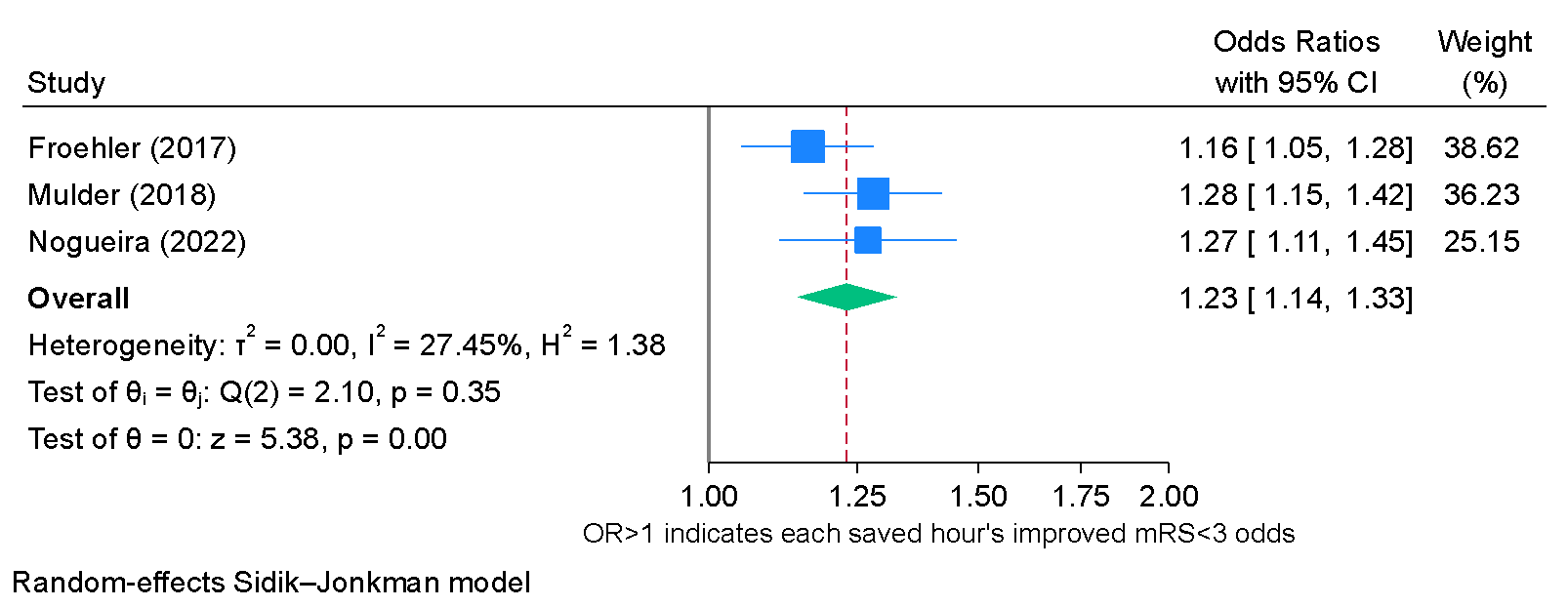


**Figure S3-10.** Forest plot for level of occlusion

**Supplement 4. GRADE and RoB2 tables**

The detailed GRADE findings for the primary endpoint (mRS 0-2) is shown in Table S4-1. Overall evidentiary quality was “low.” The results are shown for the time frame of 0-270 minutes; the later time frame’s evidentiary quality was rated similarly. A GRADE table summarizing all endpoints is shown in Table S4-2.
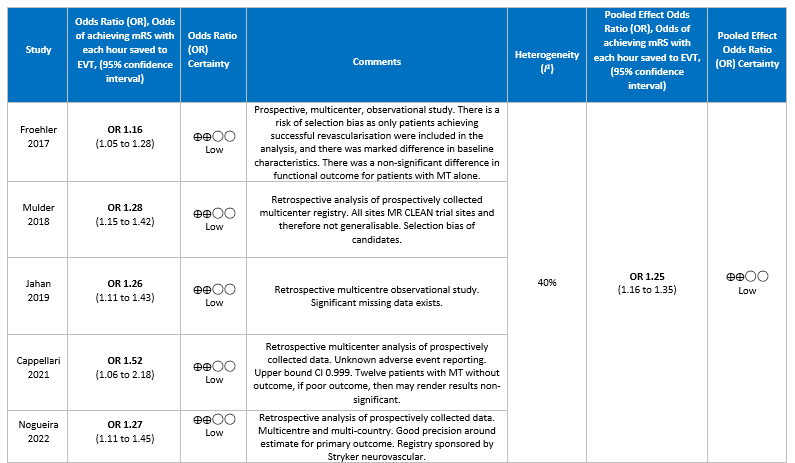


**Table S4-1.** GRADE summary for primary endpoint (mRS 0-2)


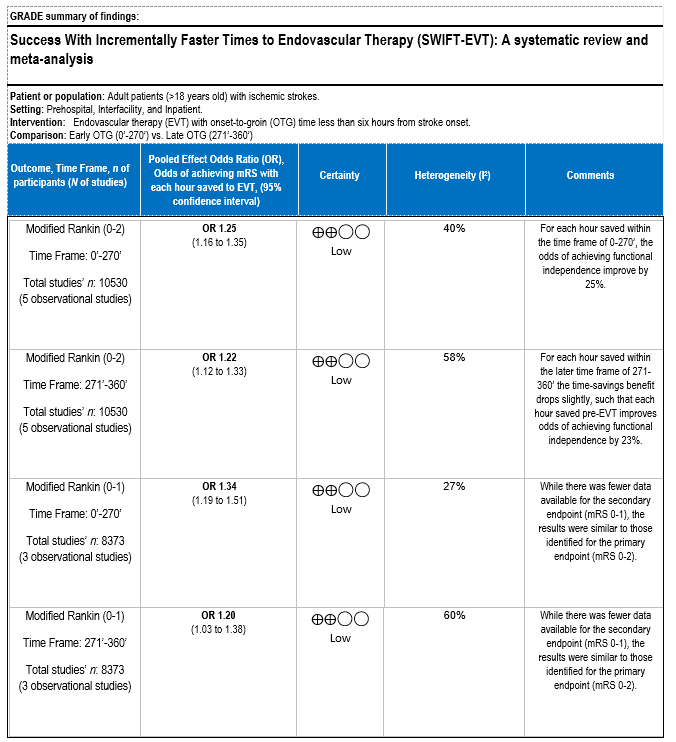


**Table S4-2.** GRADE summary table for all endpoints

# **ROBINS-I tool (Stage I): At protocol stage**

## **Specify the review question**

| Participants | Acute Ischemic Stroke (AIS) patients |
| --- | --- |
| Experimental intervention | Endovascular Therapy (EVT) |
| Comparator | Time Intervals between stroke onset-to-EVT |
| Outcomes | Functional Independence (mRS < 0-2), Excellent Outcome (mRS 0-1) |

## **List the confounding domains relevant to all or most studies**

| Interhospital transfer between primary and comprehensive stroke centers, patient prognostic factors,  qualifying NIHSS, occlusion location, use of tPA, time from onset, collateral score. |
| --- |

## **List co-interventions that could be different between intervention groups and that could impact on outcomes**

| Receival of intravenous therapy (IVtPA), mechanical thrombectomy (alone). |
| --- |

# **ROBINS-I tool (Stage II): For each study**

## **Specify a target randomized trial specific to the study - Mulder et al.**

| Design | Prospective multicenter observational single arm study of real-world MT for acute stroke |
| --- | --- |
| Participants | AIS due to large vessel occlusion patients |
| Experimental intervention | Mechanical thrombectomy |
| Comparator | Stroke onset to EVT, outcomes differences between direct and transfer patients undergoing MT, potential impact of local hospital bypass. |

## **Is your aim for this study…?**

| X | to assess the effect of *assignment to* intervention |
| --- | --- |
| ⬜ | to assess the effect of *starting and adhering to* intervention |

## **Specify the outcome**

Specify which outcome is being assessed for risk of bias (typically from among those earmarked for the Summary of Findings table). Specify whether this is a proposed benefit or harm of intervention.

| We only looked at positive endpoints of survival. Improved functional survival (mRS 0-2 at 90 days), improved independence (mRS 0-1 at 90 days), and improved survival. Proposed benefit of intervention. |
| --- |

## **Specify the numerical result being assessed**

In case of multiple alternative analyses being presented, specify the numeric result (e.g. RR = 1.52 (95% CI 0.83 to 2.77) and/or a reference (e.g. to a table, figure or paragraph) that uniquely defines the result being assessed.

| Refer to Table 2 |
| --- |

## **Specify a target randomized trial specific to the study - Nogueira et al.**

| Design | Multicentric, prospective, international, open label registry. |
| --- | --- |
| Participants | AIS patients with occlusions in the intracranial internal carotid artery or the M1 or M2 segments of the middle cerebral artery, pre-morbid mRS 0-2 and time from LSW to arterial puncture. |
| Experimental intervention | Endovascular thrombectomy early vs late window |
| Comparator | Time last seen well to arterial puncture |

## **Is your aim for this study…?**

| X | to assess the effect of *assignment to* intervention |
| --- | --- |
| ⬜ | to assess the effect of *starting and adhering to* intervention |

## **Specify the outcome**

Specify which outcome is being assessed for risk of bias (typically from among those earmarked for the Summary of Findings table). Specify whether this is a proposed benefit or harm of intervention.

| We only looked at positive endpoints of survival. Improved functional survival (mRS 0-2 at 90 days), and improved survival. Proposed benefit of intervention. |
| --- |

## **Specify the numerical result being assessed**

In case of multiple alternative analyses being presented, specify the numeric result (e.g. RR = 1.52 (95% CI 0.83 to 2.77) and/or a reference (e.g. to a table, figure or paragraph) that uniquely defines the result being assessed.

| Refer to table 2 |
| --- |

**Specify a target randomized trial specific to the study - Froehler et al.**

| Design | Prospective, multicenter, observational, single-arm study of real-world MT |
| --- | --- |
| Participants | AIS patients with acute stroke due to large vessel occlusion, treated within 8 hours of stroke onset, with a pre-stroke modified Rankin Scale (mRS) score of <2, and a pretreatment NIHSS score of >8 and <30. |
| Experimental intervention | Mechanical Thrombectomy with or without intravenous therapy |
| Comparator | Median onset-to-revascularization time |

## **Is your aim for this study…?**

| X | to assess the effect of *assignment to* intervention |
| --- | --- |
| ⬜ | to assess the effect of *starting and adhering to* intervention |

## **Specify the outcome**

Specify which outcome is being assessed for risk of bias (typically from among those earmarked for the Summary of Findings table). Specify whether this is a proposed benefit or harm of intervention.

| We only looked at positive endpoints of survival. Improved functional survival (mRS 0-2 at 90 days) and improved independence (mRS 0-1 at 90 days), and improved survival. Proposed benefit of intervention. |
| --- |

## **Specify the numerical result being assessed**

In case of multiple alternative analyses being presented, specify the numeric result (e.g. RR = 1.52 (95% CI 0.83 to 2.77) and/or a reference (e.g. to a table, figure or paragraph) that uniquely defines the result being assessed.

| Refer to figure 2 |
| --- |

## **Specify a target randomized trial specific to the study - Jahan et al.**

| Design | Retrospective cohort study using data prospectively collected |
| --- | --- |
| Participants | AIS patients with anterior circulation large vessel occlusion and last-known-well time to arterial puncture interval of 8 hours or less. |
| Experimental intervention | Endovascular-reperfusion therapy |
| Comparator | Onset (last-known-well) to arterial puncture and hospital arrival to arterial puncture |

## **Is your aim for this study…?**

| X | to assess the effect of *assignment to* intervention |
| --- | --- |
| ⬜ | to assess the effect of *starting and adhering to* intervention |

## **Specify the outcome**

Specify which outcome is being assessed for risk of bias (typically from among those earmarked for the Summary of Findings table). Specify whether this is a proposed benefit or harm of intervention.

| We only looked at positive endpoints of survival. Improved functional survival (mRS 0-2 at 90 days), improved independence (mRS 0-1 at 90 days), and improved survival. Proposed benefit of intervention. |
| --- |

## **Specify the numerical result being assessed**

In case of multiple alternative analyses being presented, specify the numeric result (e.g. RR = 1.52 (95% CI 0.83 to 2.77) and/or a reference (e.g. to a table, figure or paragraph) that uniquely defines the result being assessed.

| Refer to table 2 |
| --- |

## **Specify a target randomized trial specific to the study - Capillari et al.**

| Design | Prospectively collected data |
| --- | --- |
| Participants | AIS patients receiving IVT and/or thrombectomy |
| Experimental intervention | IVT and thrombectomy |
| Comparator | Onset-to-door time, door-to-needle, onset-to-needle |

## **Is your aim for this study…?**

| X | to assess the effect of *assignment to* intervention |
| --- | --- |
| ⬜ | to assess the effect of *starting and adhering to* intervention |

## **Specify the outcome**

Specify which outcome is being assessed for risk of bias (typically from among those earmarked for the Summary of Findings table). Specify whether this is a proposed benefit or harm of intervention.

| We only looked at positive endpoints of survival. Improved independence (mRS 0-1 at 90 days) and improved functional survival (mRS 0-2 at 90 days), and improved survival. Proposed benefit of intervention. |
| --- |

## **Specify the numerical result being assessed**

In case of multiple alternative analyses being presented, specify the numeric result (e.g. RR = 1.52 (95% CI 0.83 to 2.77) and/or a reference (e.g. to a table, figure or paragraph) that uniquely defines the result being assessed.

| Refer to table 1. |
| --- |

**Preliminary consideration of confounders**

Complete a row for each important confounding domain (i) listed in the review protocol; and (ii) relevant to the setting of this particular study, or which the study authors identified as potentially important.

#### *“Important” confounding domains are those for which, in the context of this study, adjustment is expected to lead to a clinically important change in the estimated effect of the intervention. “Validity” refers to whether the confounding variable or variables fully measure the domain, while “reliability” refers to the precision of the measurement (more measurement error means less reliability).*

| **(i) Confounding domains listed in the review protocol** | | | | |
| --- | --- | --- | --- | --- |
| **Confounding domain** | **Measured variable(s)** | **Is there evidence that controlling for this variable was unnecessary?*** | **Is the confounding domain measured validly and reliably by this variable (or these variables)?** | **OPTIONAL: Is failure to adjust for this variable (alone) expected to favour the experimental intervention or the comparator?** |
| Transfer status: Interhospital transfer between primary and comprehensive stroke centres | Onset-to-puncture time | No | Yes |  |
| Patient prognostic factors | Age, sex, medical history, pre-stroke mRS scores, ASPECTS and qualifying NIHSS scores at baseline, receival of general anesthesia | Yes (Refer to the discussion section of Mulder et al.) | Yes |  |
| Occlusion location | Computed tomography (CT), magnetic resonance imaging (MR) to determine if occlusion is located in the internal carotid artery or middle cerebral artery (M1 or M2) | No | Yes |  |
| Use of tPA | tPA administration | No | Yes |  |
| Time from onset | Time from stroke onset to arterial puncture (EVT) | No | Yes |  |
| Collateral score | Collateral assessment based on Collateral Score (CS) system (refer to Mulder) | No | Yes |  |

## **Preliminary consideration of co-interventions**

Complete a row for each important co-intervention (i) listed in the review protocol; and (ii) relevant to the setting of this particular study, or which the study authors identified as important.

#### *“Important” co-interventions are those for which, in the context of this study, adjustment is expected to lead to a clinically important change in the estimated effect of the intervention.*

| **(i) Co-interventions listed in the review protocol** | | |
| --- | --- | --- |
| **Co-intervention** | **Is there evidence that controlling for this co-intervention was unnecessary (e.g. because it was not administered)?** | **Is presence of this co-intervention likely to favour outcomes in the experimental intervention or the comparator** |
| Receival of intravenous therapy (IV-tPA) | No. Patients who receive IvTPA for up to 4.5 hours after symptoms onset increases likelihood of recovery to independence (Capellari et al.) | Favours patients who had received intravenous treatment before 4.5 hours of symptoms onset |
| Mechanical Thrombectomy (MT) alone | No. Comparison of outcomes by mRS shift analysis showed significant difference between transfer and direct groups for MT alone (Froehler) | Favour patients who had MT alone |

**Risk of bias assessment**

Responses underlined in green are potential markers for low risk of bias, and responses in red are potential markers for a risk of bias. Where questions relate only to sign posts to other questions, no formatting is used.

|  | **Signalling questions** | **Description** | **Response options** |
| --- | --- | --- | --- |
| **Bias due to confounding** | | | |
|  | 1.1 Is there potential for confounding of the effect of intervention in this study?  **If N/PN to 1.1:** the study can be considered to be at low risk of bias due to confounding and no further signaling questions need be considered |  | N |
|  | **If Y/PY to 1.1**: determine whether there is a need to assess time-varying confounding: |  |  |
|  | 1.2. Was the analysis based on splitting participants’ follow up time according to intervention received?  **If N/PN**, answer questions relating to baseline confounding (1.4 to 1.6)  **If Y/PY**, go to question 1.3. | Participants were divided into cohorts based on their onset-to-groin puncture time. No participants could switch between cohorts. | N |
|  | 1.3. Were intervention discontinuations or switches likely to be related to factors that are prognostic for the outcome?  **If N/PN**, answer questions relating to baseline confounding (1.4 to 1.6)  **If Y/PY**, answer questions relating to both baseline and time-varying confounding (1.7 and 1.8) | N/A | N/A |

|  | **Questions relating to baseline confounding only** | | |
| --- | --- | --- | --- |
|  | 1.4. Did the authors use an appropriate analysis method that controlled for all the important confounding domains? | Study quality was assessed using standardized tools and ROBINS-I and GRADE tables were used to analyze the study’s risk of bias. Applied a Hartung-Knapp-Sidik-Jonkman (HKSJ) RE approach recommended for small-*N* MA. A second RE approach, the DerSimonian-Laird (DL) model was used to assess sensitivity to model selection. A third modeling approach, fixed-effect (FE) modeling, was executed solely as an informal indicator of study heterogeneity. | Y |
|  | 1.5. **If Y/PY to 1.4**: Were confounding domains that were controlled for measured validly and reliably by the variables available in this study? | Refer to statistical analysis and reporting section of methods section of manuscript. | Y |
|  | 1.6. Did the authors control for any post-intervention variables that could have been affected by the intervention? | mRS values were reported and utilized in calculations directly as reported from included studies. Other clinical outcomes such as mortality were also reported directly from the included studies. | N |
|  | **Questions relating to baseline and time-varying confounding** | |  |
|  | 1.7. Did the authors use an appropriate analysis method that controlled for all the important confounding domains and for time-varying confounding? | Refer to statistical analysis and reporting section of methods section of manuscript | Y |
|  | 1.8. **If Y/PY to 1.7**: Were confounding domains that were controlled for measured validly and reliably by the variables available in this study? | Refer to statistical analysis and reporting section of methods section of manuscript | Y |
|  | **Risk of bias judgement** | The overall risk of bias was “low.” | Low |
|  | Optional: What is the predicted direction of bias due to confounding? |  | N/A |

| **Bias in selection of participants into the study** | | | |
| --- | --- | --- | --- |
|  | 2.1. Was selection of participants into the study (or into the analysis) based on participant characteristics observed after the start of intervention?  **If N/PN to 2.1:** go to 2.4 | Selection of the participants in the study was based on the six hours to EVT start time window. | N |
|  | 2.2. **If Y/PY to 2.1**: Were the post-intervention variables that influenced selection likely to be associated with intervention?  2.3 **If Y/PY to 2.2**: Were the post-intervention variables that influenced selection likely to be influenced by the outcome or a cause of the outcome? |  | NA  NA |
|  | 2.4. Do start of follow-up and start of intervention coincide for most participants? | Follow-up for mRS scores and mortality were recorded at 90 days post EVT. | Y |
|  | 2.5. **If Y/PY to 2.2 and 2.3, or N/PN to 2.4**: Were adjustment techniques used that are likely to correct for the presence of selection biases? | N/A | N/A |
|  | **Risk of bias judgement** | The overall risk of bias was “low.” | Low |
|  | Optional: What is the predicted direction of bias due to selection of participants into the study? |  | N/A |

| **Bias in classification of interventions** | | | |
| --- | --- | --- | --- |
|  | 3.1 Were intervention groups clearly defined? | Patients were split up into clear distinct time frames based on their reported time intervals in the included studies. | Y |
|  | 3.2 Was the information used to define intervention groups recorded at the start of the intervention? | Intervention group was defined as patients who had received EVT within 6 hours from onset of symptoms. Refer to the methods section of the manuscript for additional information. | Y |
|  | 3.3 Could classification of intervention status have been affected by knowledge of the outcome or risk of the outcome? | EVT under 6 hours is a widely accepted window for thrombectomy treatment. All studies above 6 hours were excluded. | N |
|  | **Risk of bias judgement** | The overall risk of bias was “low.” | Low |
|  | Optional: What is the predicted direction of bias due to classification of interventions? |  | N/A |

| **Bias due to deviations from intended interventions** | | | |
| --- | --- | --- | --- |
|  | **If your aim for this study is to assess the effect of assignment to intervention, answer questions 4.1 and 4.2** | |  |
|  | 4.1. Were there deviations from the intended intervention beyond what would be expected in usual practice? | No deviations from intended intervention were recorded. | N |
|  | 4.2. **If Y/PY to 4.1**: Were these deviations from intended intervention unbalanced between groups *and* likely to have affected the outcome? | N/A | N/A |
|  | **If your aim for this study is to assess the effect of starting and adhering to intervention, answer questions 4.3 to 4.6** | |  |
|  | 4.3. Were important co-interventions balanced across intervention groups? | N/A | N/A |
|  | 4.4. Was the intervention implemented successfully for most participants? | N/A | N/A |
|  | 4.5. Did study participants adhere to the assigned intervention regimen? | N/A | N/A |
|  | 4.6. **If N/PN to 4.3, 4.4 or 4.5**: Was an appropriate analysis used to estimate the effect of starting and adhering to the intervention? | N/A | N/A |
|  | **Risk of bias judgement** | The overall risk of bias was “low.” | Low |
|  | Optional: What is the predicted direction of bias due to deviations from the intended interventions? |  | N/A |

| **Bias due to missing data** | | | |
| --- | --- | --- | --- |
|  | 5.1 Were outcome data available for all, or nearly all, participants? |  | Y |
|  | 5.2 Were participants excluded due to missing data on intervention status? |  | N |
|  | 5.3 Were participants excluded due to missing data on other variables needed for the analysis? |  | N |
|  | 5.4 **If PN/N to 5.1, or Y/PY to 5.2 or 5.3**: Are the proportion of participants and reasons for missing data similar across interventions? | N/A | N/A |
|  | 5.5 **If PN/N to 5.1, or Y/PY to 5.2 or 5.3**: Is there evidence that results were robust to the presence of missing data? | N/A | N/A |
|  | **Risk of bias judgement** | The overall risk of bias was “low.” | Low |
|  | Optional: What is the predicted direction of bias due to missing data? |  | N/A |

| **Bias in measurement of outcomes** | | | |
| --- | --- | --- | --- |
|  | 6.1 Could the outcome measure have been influenced by knowledge of the intervention received? | N/A | N |
|  | 6.2 Were outcome assessors aware of the intervention received by study participants? |  | Y |
|  | 6.3 Were the methods of outcome assessment comparable across intervention groups? |  | Y |
|  | 6.4 Were any systematic errors in measurement of the outcome related to intervention received? |  | N |
|  | **Risk of bias judgement** | The overall risk of bias was “low.” | Low |
|  | Optional: What is the predicted direction of bias due to measurement of outcomes? |  | Favours experimental / Favours comparator / Towards null /Away from null / Unpredictable |

| **Bias in selection of the reported result** | | | |
| --- | --- | --- | --- |
|  | Is the reported effect estimate likely to be selected, on the basis of the results, from... |  |  |
|  | 7.1. ... multiple outcome *measurements* within the outcome domain? |  | N |
|  | 7.2 ... multiple *analyses* of the intervention-outcome relationship? |  | N |
|  | 7.3 ... different *subgroups*? |  | N |
|  | **Risk of bias judgement** | The overall risk of bias was “low.” | Low |
|  | Optional: What is the predicted direction of bias due to selection of the reported result? |  | N/A |

| **Overall bias** | | | |
| --- | --- | --- | --- |
|  | **Risk of bias judgement** | The overall risk of bias was “low.” | Low |
|  | Optional: What is the overall predicted direction of bias for this outcome? |  | N/A |

**Table S4-3.** RoB2 evidence summary tables
